# Metabolic Outputs of the Gut Microbiome: Implications for Epilepsy

**DOI:** 10.3390/cells15161492

**Published:** 2026-08-19

**Authors:** Allison Gallucci, Xi Guo, Devika Shukla, Susan L. Campbell

**Affiliations:** 1 Graduate Program in Translational Biology Medicine and Health, Virginia Tech, Roanoke, VA 24016, USA; allisongallucci@gmail.com; 2 Cell, Development and Integrative Biology, University of Alabama at Birmingham, Birmingham, AL 35294, USA; xguo2@uab.edu; 3 Neuroscience Graduate Program, University of Miami Miller School of Medicine, Miami, FL 33136, USA; dxs4028@miami.edu

**Keywords:** epilepsy, gut microbiome, metabolites, seizures, gut–brain axis

## Abstract

**Highlights:**

**What are the main findings?**

- Microbiota-derived metabolites function as critical cellular mediators of gut–brain communication, influencing neurotransmission, neuroinflammatory signaling, and blood–brain barrier integrity.
- Emerging evidence identifies multiple microbiota-derived molecules, including neurotransmitters, vitamins, and polyphenol metabolites, as key modulators of neuronal hyperexcitability and seizure-related pathways.

**What are the implications of the main findings?**

- Understanding how microbial metabolites regulate cellular processes in the brain may reveal previously unrecognized mechanisms underlying epileptogenesis.
- Microbiome-targeted interventions designed to restore beneficial metabolite production have the potential to become a new class of precision therapies for epilepsy and other neurological disorders.

**Abstract:**

Background: Microbiome-based mechanisms have emerged as a key area of investigation in epilepsy, given the growing recognition that gut microbial communities can modulate central nervous system (CNS) function through the gut–brain axis. Epilepsy is a common chronic neurological disorder affecting more than 65 million people worldwide, and despite the availability of anti-seizure medications (ASMs), approximately 30% of patients develop drug-resistant epilepsy. Current ASMs primarily suppress seizures rather than prevent disease progression, highlighting the need for alternative therapeutic strategies. In this context, increasing evidence supports a role for microbiota-dependent pathways in modulating seizure activity and treatment responsiveness. However, the mechanistic basis of these interactions remains incompletely understood. Methods: This narrative review synthesizes findings from the existing literature to examine the role of microbiota-derived metabolites, including neurotransmitters, vitamins, and the polyphenol metabolite S-equol, in gut–brain communication relevant to epilepsy. Evidence was drawn from both preclinical animal models and clinical studies to provide an integrated, mechanistic perspective on how these pathways may influence central nervous system function and seizure susceptibility. Emphasis was placed on studies describing molecular, metabolic, and signaling mechanisms linking the gut microbiome to epileptogenesis and treatment response. Results: Current evidence indicates that communication between the gut and CNS occurs through neural pathways, such as the vagus nerve, as well as through circulating microbial metabolites. These metabolites can cross the intestinal barrier and, in some cases, the blood–brain barrier (BBB), serving as key mediators of host–microbiota signaling. Emerging studies suggest that while some microbial metabolites may directly influence neuronal hyperexcitability and seizure susceptibility, others likely exert secondary or modulatory effects through broader metabolic and immune pathways. However, the precise mechanisms underlying these interactions remain incompletely understood. Conclusions: Some microbial-derived metabolites may serve as promising biomarkers and mechanistic mediators of epilepsy; however, further investigation is needed to define the molecular and cellular pathways through which these metabolites influence seizure susceptibility and epileptogenesis.

## 1. Introduction

Epilepsy is a chronic and disabling neurological disorder affecting over 65 million people worldwide and is associated with significant social, psychological, cognitive, and neurobiological burdens, as well as an increased risk of mortality [1,2,3,4]. Its defining feature is the occurrence of recurrent, unprovoked seizures arising from transient episodes of abnormal, excessive, and hypersynchronous neuronal firing. Epilepsy is a heterogeneous condition with diverse etiologies, including genetic variants, developmental abnormalities, brain tumors, trauma, autoimmune dysfunction, and infections of the central nervous system (CNS) [5,6]. At the cellular level, seizures reflect disruptions in the balance between excitatory glutamatergic and inhibitory γ-aminobutyric acid (GABA)ergic signaling [7]. However, growing evidence indicates that epilepsy extends beyond a purely neuron-centered disorder. Glial dysfunction, immune activation, extracellular matrix alterations, and systemic inflammatory and metabolic disturbances all contribute to seizure susceptibility and disease progression [8,9,10,11]. Indeed, CNS infections remain a major global cause of epilepsy, and neuroinflammation is a consistent feature across human epilepsy and multiple animal models [12,13,14].

Despite the availability of antiseizure medications (ASMs), approximately 30% of patients remain drug resistant, a proportion that has changed little despite the introduction of new therapeutic agents [15,16,17]. This persistent treatment gap underscores the need for novel therapeutic strategies that extend beyond CNS-restricted targets. Increasing recognition that peripheral systems, including the gut microbiome, influence seizure susceptibility has opened new avenues for understanding epileptogenesis [18,19]. The gut microbiome has emerged as a key regulator of host neurobiology through bidirectional communication along the gut–brain axis. Microbial communities influence neuronal excitability through multiple mechanisms, including immune modulation, vagal signaling, neurotransmitter production, and the generation of bioactive metabolites [20]. Consistent with this, alterations in gut microbial composition have been associated with seizure susceptibility, drug-resistant epilepsy, and differential treatment responses. Notably, the clinical efficacy of the ketogenic diet (KD), a well-established therapy for refractory epilepsy, has been shown to depend, in part, on microbiome-mediated mechanisms [21]. Moreover, ASMs themselves can alter gut microbial composition [22], raising the possibility that some drug effects, or variability in responsiveness, may be mediated through microbiome-dependent pathways.

As the field shifts from descriptive analyses of microbial composition toward functional insights, microbiome-derived metabolites have gained particular attention. Metabolites such as short-chain fatty acids (SCFAs), tryptophan derivatives, bile acids, and phytoestrogen metabolites such as S-equol can modulate neuroinflammation, blood–brain barrier (BBB) integrity, neurotransmitter systems, and synaptic plasticity [23]. These mechanisms are directly relevant to epilepsy, as they influence excitatory–inhibitory balance, glial activation, and neuronal network stability. Importantly, metabolite-based mechanisms offer practical advantages, as they are quantifiable in biological fluids, represent defined molecular pathways, and can be modulated through diet, prebiotics, probiotics, or pharmacological interventions. Collectively, these findings highlight the emerging importance of metabolite-mediated mechanisms in epilepsy research, an area still being actively elucidated. Identifying specific microbial metabolites that influence seizure thresholds and disease trajectories may facilitate the development of next-generation therapeutic strategies targeting the gut–brain axis.

## 2. Literature Search Methods

This narrative review was informed by a literature search conducted from database inception through July 2026. Relevant literature was identified through searches of PubMed, Web of Science, and Google Scholar using combinations of search terms including “gut microbiome”, “gut microbiota”, “microbiota-derived metabolites”, “vitamins”, “neurotransmitters”, “polyphenol S-equol”, “lactate”, “short-chain fatty acids”, “gut-brain axis”, “signaling pathway”, “neuronal excitability”, “seizures” and “epilepsy.” Additional studies were identified through examination of the reference lists of relevant publications. Evidence was drawn from preclinical animal and cellular models, clinical studies and selected foundational reviews, with emphasis placed on studies describing molecular, metabolic, and signaling mechanisms linking microbiota-derived molecules to seizure susceptibility and epileptogenesis. Studies were selected based on their relevance to microbial metabolite production, gut–brain communication, neuronal function, and epilepsy-related outcomes, with priority given to original research articles and mechanistic investigations. Only peer-reviewed publications were considered; conference abstracts and preprints were excluded. Consistent with the narrative review approach, no formal risk-of-bias assessment or qualitative synthesis was conducted.

The literature included in this review was organized according to the major classes of microbiota-derived metabolites discussed in this review, including neurotransmitter-related metabolites, short-chain fatty acids, lactate, vitamins and the polyphenol-derived metabolite S-equol. For each class of metabolite, evidence was organized according to the primary mechanisms through which these metabolites influence gut–brain communication, including modulation of neurotransmission, blood–brain barrier integrity, and neuronal excitability. Where applicable findings were further categorized according to the level of evidence available, distinguishing experimental from human studies.

## 3. The Gut Microbiome and Epilepsy

### 3.1. Composition and Function of the Gut Microbiome

The human gastrointestinal tract harbors a complex and dynamic ecosystem of microorganisms that profoundly influence host physiology. The term gut microbiota refers to the diverse microorganisms inhabiting the body, whereas the gut microbiome encompasses these organisms along with their collective genetic material. Microbial colonization begins at birth and evolves throughout early childhood before stabilizing in adulthood [24]. Early-life microbial communities are shaped by environmental factors such as mode of delivery, with vaginal and cesarean births exposing infants to distinct microbial populations [25]. Additional influences, including antibiotics, medications, genetics, diet, and environmental exposures, continue to shape gut microbial composition across the lifespan [26]. The gut microbiome performs a wide range of essential functions. These include fermenting indigestible dietary components into bioactive metabolites, synthesizing vitamins, detoxifying harmful compounds, preventing pathogen colonization, reinforcing intestinal barrier integrity, and modulating innate and adaptive immune responses [27]. Through these activities, microbial communities establish a symbiotic relationship with the host. Disruptions in microbial composition or diversity, collectively termed dysbiosis, have been linked to numerous disease states, including obesity, inflammatory bowel disease, cardiovascular disorders, and neurological conditions such as epilepsy [28].

### 3.2. Evidence Linking Gut Microbiota to Epilepsy

Growing evidence indicates that the gut microbiota plays a significant role in the development and modulation of epilepsy [29]. Clinical data demonstrate that individuals with epilepsy often exhibit distinct gut microbial profiles, including reduced levels of beneficial genera such as *Lactobacillus* [30]. These observations raise the possibility that microbial alterations contribute to seizure susceptibility or disease progression. Consistent with this notion, gut dysbiosis has been repeatedly associated with drug-resistant epilepsy [31,32,33], and non-responders to ASMs display distinct microbial signatures compared with both healthy controls and treatment-responsive patients [34,35]. Experimental models further support a causal relationship between the gut microbiome and seizure activity. For example, in kainic acid (KA)-induced seizure models, rats exhibit oxidative stress, inflammation, cognitive deficits, and elevated glutamate release, all of which are partially ameliorated by prebiotic, probiotic, or synbiotic interventions [36]. In genetic epilepsy models, such as Dravet syndrome caused by *Scn1a* mutations, gut dysbiosis has been reported, and specific microbial taxa correlate with disease severity [37]. In these models, increased Clostridium and decreased Romboutsia abundance are associated with more severe phenotypes, whereas KD-induced shifts toward increased Firmicutes and decreased Bacteroidetes correlate with reduced seizure frequency and duration.

Additional studies demonstrate that microbiota manipulation directly alters seizure susceptibility. In rodent models of absence epilepsy, fecal microbiota transplantation from healthy donors reduces seizure activity [38]. Similarly, transplantation of microbiota from control animals decreases seizure frequency in stress-exposed animals, whereas microbiota from stressed donors increases seizure susceptibility in otherwise healthy recipients [39]. Consistent with these findings, control mice receiving microbiota from epileptic donors are more prone to develop status epilepticus following a subclinical dose of pilocarpine [40]. Collectively, these findings support a bidirectional relationship between the gut microbiota and epilepsy, in which microbial composition influences seizure susceptibility and disease outcomes. However, further mechanistic studies are required to establish causality and define the pathways involved.

### 3.3. Nutrition, Gut Bacteria, and Seizure Outcomes

Dietary interventions have long occupied a central position in the therapeutic landscape of epilepsy, providing a compelling example of how systemic metabolic shifts can reverberate across the brain–gut–microbiome axis. Early clinical observations in 1911 revealed that fasting reduces seizure frequency [41], ultimately leading to the development of the KD in 1921 [42]. This high-fat, low-carbohydrate regimen induces a metabolic state characterized by enhanced fatty acid oxidation and elevated ketone body production, but its effects extend well beyond host metabolism to reshape the intestinal microbial ecosystem [43,44]. By promoting fat utilization and increasing circulating ketone bodies, the KD induces systemic metabolic changes alongside marked alterations in gut microbial composition. A growing body of evidence from both clinical cohorts and experimental models indicates that ketogenic therapy reproducibly alters gut microbial composition [21,45,46,47], with consistent shifts observed in children with drug-resistant epilepsy [48,49,50,51,52]. These changes are not merely correlative. In a landmark study employing germ-free and antibiotic-treated mouse models, Olson et al. demonstrated that the antiseizure efficacy of the KD depends on the presence of gut microbiota [21]. Specifically, colonization of gnotobiotic mice with KD-associated microbial communities, *Akkermansia* and *Parabecterioides*, was sufficient to confer seizure protection in chemically induced seizure paradigms, including those using pentylenetetrazole (PTZ). Mechanistically, this effect was linked to alterations in systemic metabolite pools, most notably reductions in circulating γ-glutamylated amino acids, and a shift in central neurotransmitter balance toward enhanced GABAergic tone relative to glutamatergic excitation. Such findings underscore the capacity of microbiota-derived metabolites to influence neuronal excitability, consistent with the broader principle that microbial products, including neurotransmitter precursors, can modulate host neural circuits.

At the microbial level, KDs have been associated with enrichment of specific taxa implicated in neurometabolic signaling. For instance, increases in *Akkermansia muciniphila* and *Parabacteroides* species have been reported in KD-fed mice, taxa that appear necessary for seizure protection in transplantation studies [21]. More broadly, bacteria capable of producing neuroactive metabolites, including GABA-producing genera such as *Bifidobacterium*, *Lactobacillus*, and *Bacteroides*, are increasingly recognized as functional mediators within the gut–brain–microbiome axis. These organisms synthesize GABA primarily via glutamate decarboxylase pathways, with exported GABA capable of acting locally within the enteric nervous system or indirectly influencing brain function through vagal, immune, or metabolic routes.

Probiotic-based interventions provide further mechanistic support for a microbiota–seizure axis. In rodent models of chemically induced seizures, including PTZ-kindling paradigms, administration of GABA-producing probiotics reduces seizure severity and improves behavioral outcomes [53,54]. These effects are associated with increased brain GABA levels and reductions in pro-oxidative mediators such as nitric oxide. Although the mechanisms responsible for these changes remain incompletely understood, they may involve modulation of host GABAergic signaling pathways, vagal nerve communication and neuroinflammatory processes, suggesting convergence on inhibitory neurotransmission and neuroimmune pathways. Clinical studies, although more limited in scale, similarly report reductions in seizure frequency in patients with refractory epilepsy receiving probiotic supplementation [55,56], alongside improvements in comorbid anxiety and depressive symptoms, which are consistent with the pleiotropic effects of microbiota-derived neuromodulators.

Dietary modulation of specific metabolic pathways further illustrates the mechanistic interplay between nutrition, microbiota, and seizure susceptibility. Tryptophan metabolism is of relevance, as it intersects with both serotonergic and microbial signaling networks. In murine models of audiogenic seizures, high-tryptophan diets reduce seizure-induced respiratory arrest [57], while simultaneously altering gut microbial composition, including increases in Proteobacteria and Actinobacteria [58]. These findings suggest that diet-induced shifts in microbial ecology can recalibrate neuroactive metabolite production, thereby influencing brainstem circuits governing respiratory and seizure thresholds.

Clinical observations also point toward species-level microbial changes associated with therapeutic response. In infants with epileptic spasms treated with the KD, reductions in seizure frequency coincide with increased abundance of *Streptococcus thermophilus* and *Lactococcus lactis* [59], taxa with known metabolic activity in amino acid fermentation and potential contributions to neurotransmitter precursor pools.

Collectively, these findings support a model in which dietary interventions exert antiseizure effects through coordinated modulation of host metabolism and the gut microbiota. Microbial metabolites, including GABA, SCFAs, and amino acid derivatives, act as intermediaries within this network, influencing neuronal excitability through microbial-associated pathways. This integrated framework not only refines our understanding of ketogenic and probiotic therapies but also highlights the microbiome as a tractable target for adjunctive interventions in epilepsy.

## 4. Mechanistic Pathways Connecting the Gut to the CNS

The emerging evidence linking dietary interventions, microbial community structure, and seizure outcomes naturally converges on a central question: through which biological conduits do signals originating in the gut influence neuronal excitability within the CNS? The answer lies within the intricate architecture of the gut–brain axis, a multidimensional communication network that integrates neural, endocrine, immune, and metabolic signaling into a unified regulatory system.

**Figure 1:**
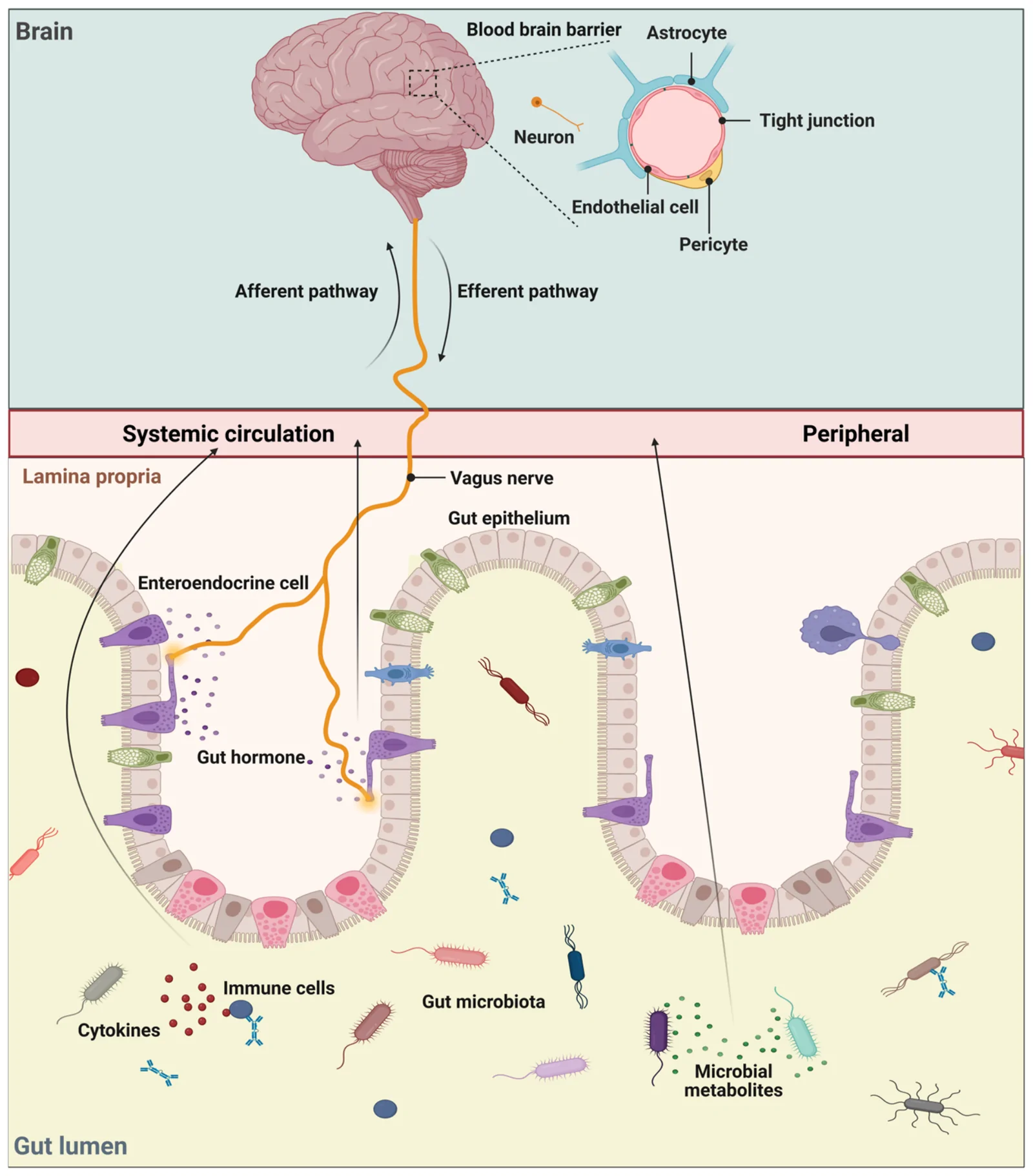
Pathways through which the gut microbiota communicates with the central nervous system. Gut microbes modulate immune cells, stimulating the production of cytokines that enter circulation and signal to the brain. Microbial interactions with enteroendocrine cells generate neuroactive molecules that activate vagal pathways projecting to the CNS. In addition, gut bacteria produce neurotransmitters and metabolites that can circulate systemically and influence brain function. Created in BioRender. Guo, X. (2026); https://biorender.com/0nvqyg2 (accessed on 21 July 2026).

This axis operates not as a linear pathway but as a dynamic and reciprocal interface, wherein the gastrointestinal tract, its resident microbiota, and the CNS engage in continuous biochemical dialogue. Signals are transmitted along convergent routes, including vagal and spinal neural circuits, circulating metabolites and hormones, immune mediators, and neuroendocrine outputs governed by the hypothalamic–pituitary–adrenal (HPA) axis. These pathways are further modulated by a diverse repertoire of microbiota-derived metabolites, most notably SCFAs, neurotransmitters, and amino acid derivatives, that function as chemical intermediaries bridging microbial activity with host physiology (Figure 1).

### 4.1. Vagal Signaling Pathway

The gut microbiome influences CNS function through both direct neural routes and indirect metabolic and immune pathways. Among these, the vagus nerve represents a principal conduit for rapid bidirectional communication between the gut and the brain. This cranial nerve integrates visceral sensory input with central autonomic processing and plays a key role in regulating physiological processes such as pain perception, thermoregulation, gastrointestinal motility, and cardiovascular function [60]. Anatomically, the vagus comprises both efferent (motor) and afferent (sensory) fibers that connect the brainstem to multiple regions of the gastrointestinal tract. Vagal efferents originate from neuronal cell bodies located in the dorsal motor nucleus of the vagus (DMNV) and the nucleus ambiguus, where they modulate gastrointestinal motility and cardiovascular activity [61]. In contrast, vagal afferent neurons reside in the nodose ganglia and project centrally to the DMNV. Together, the NTS and DMNV form the dorsal vagal complex, a critical integrative hub that coordinates sensory input from the gut with autonomic and neuroendocrine responses [61].

Within this circuitry, vagal afferents are uniquely positioned to transduce signals arising from the intestinal milieu, including those shaped by the gut microbiota. These signals may be initiated by neurotransmitters and metabolites released by enteroendocrine cells (EECs), such as serotonin (5-HT) and SCFAs, or by diffusible metabolites that cross the intestinal epithelium and interact directly with vagal terminals [62]. Notably, vagal afferents express receptors capable of sensing microbiota-derived signals, including free fatty acid receptors (FFAR) 2 and FFAR 3, positioning the vagus nerve as a key interface through which microbial metabolites and microbiota-induced enteroendocrine signaling influence central physiology [63].

At the synaptic level, glutamate serves as the principal neurotransmitter mediating the transmission of vagal afferent signals to second-order neurons in the NTS. From this region, information is relayed to higher-order brain regions involved in autonomic regulation, emotional processing, and seizure susceptibility. Through reciprocal efferent projections, the CNS can also modulate gut physiology, influencing motility, secretion, and even microbial composition. This bidirectional architecture underscores the capacity of the brain to both “sense” and actively shape the gut environment.

The functional importance of this pathway is highlighted by experimental studies in which disruption of vagal signaling, such as through surgical vagotomy, abolishes many microbiota-induced effects on brain function and behavior, particularly effects related to anxiety, stress and emotional processing, indicating that intact vagal pathways are essential for a wide range of gut–brain interactions [63,64,65]. Conversely, therapeutic modulation of this circuit has demonstrated clinical efficacy. Vagus nerve stimulation, an FDA-approved intervention for drug-resistant epilepsy, reduces seizure frequency in both adult and pediatric populations [66,67]. Although the precise mechanisms remain incompletely defined, stimulation of vagal afferents is known to alter central neurotransmitter systems, including glutamatergic and GABAergic signaling, thereby shifting the balance of neural excitation and inhibition in seizure-relevant circuits [66,67]. Taken together, these findings highlight the vagus nerve as a critical mechanistic bridge linking gut-derived signals to central neural networks. By integrating microbial, metabolic, and neurochemical inputs, this pathway provides a plausible substrate through which microbiome alterations, such as those induced by diet or probiotic interventions, can modulate neuronal excitability and influence seizure outcomes.

### 4.2. Immune-Mediated Pathways

The gut microbiota plays a central role in the development and function of brain-resident immune cells, including microglia, astrocytes, and both innate and adaptive immune populations, and is therefore essential for maintaining balanced host immune responses [68]. Extensive evidence supports the immune system as a critical component of gut–brain communication [69]. Within the intestine, microbial communities regulate immune activity by shaping the function of both immune and non-immune cells locally and at distant sites throughout the body [70]. Activation of intestinal immune cells in response to microbial signals, dysbiosis, or barrier disruption leads to the release of pro- and anti-inflammatory cytokines, which can circulate systemically and influence CNS function [71].

Following CNS injury peripheral immune responses become rapidly engaged. For instance, circulating monocytes, macrophages, and T cells can infiltrate the CNS and disrupt the BBB, which is a well-established pathological feature in epilepsy [18]. Once inside the CNS, monocytes may differentiate into macrophages and, in some cases, acquire microglia-like characteristics [72]. Gut-driven inflammatory responses have been reported across several neurological conditions, including epilepsy [73]. To investigate causal interactions between gut microbes and CNS immune processes, germ-free mouse models are frequently used. These animals are raised without a microbiota and exhibit profound immune deficiencies and increased susceptibility to infection, underscoring the essential role of the microbiome in immune development [74,75].

Globally, CNS infection remains one of the leading causes of epilepsy, highlighting the strong link between immune dysregulation and epileptogenesis [4]. Importantly, inflammation also contributes to non-infectious forms of epilepsy. For example, reactive astrogliosis, a hallmark of CNS inflammation, has been shown to independently induce seizures in mice [10]. Additional evidence comes from the Theiler’s murine encephalomyelitis virus (TMEV) epilepsy model, where in response to infection infiltrating macrophages release high levels of interleukin 6 (IL-6) and tumor necrosis factor-alpha (TNF-α), promoting acute seizure development [76,77]. Immune cell infiltration such as lymphocytes and monocytes is further observed after status epilepticus, a serious condition defined as a prolonged seizure lasting ≥5 min, supporting a central role for inflammation in seizure generation [12,78]. Modulating the gut microbiome may also alter seizure susceptibility through immune-mediated pathways. For instance, the KD reshapes gut microbial communities in ways that promote anti-inflammatory effects [45], which may contribute to its therapeutic benefits, particularly in refractory epilepsy. Collectively, these findings support a mechanistic link between the gut, the immune system, and seizure susceptibility, highlighting the gut–immune axis as a critical contributor to epilepsy pathophysiology.

### 4.3. The Neuroendocrine Pathways

The gut–brain axis is also shaped by slower neuroendocrine communication networks that integrate microbial cues with systemic hormonal responses. These pathways operate through bidirectional interactions between the CNS and the gastrointestinal tract, with the HPA axis serving as a central regulatory node. In this context, the gut microbiome functions as both a modulator and a target of neuroendocrine signaling, thereby extending its influence from local intestinal processes to global physiological states.

At the interface of this communication system lies a specialized population of EECs, dispersed throughout the gastrointestinal mucosa. Although they constitute less than 1% of the epithelial cell population, their functional impact is disproportionately large due to their capacity to sense luminal contents and secrete a wide array of bioactive hormones [79]. These cells are most abundant in the proximal intestine, particularly the duodenum, with decreasing density toward the colon. EECs express diverse receptors capable of detecting microbial metabolites, nutrients, and mechanical stimuli, enabling them to act as transducers of environmental signals into endocrine outputs. Among the hormones secreted by EECs are cholecystokinin, gastric inhibitory polypeptide, and glucagon-like peptide-1, each of which exerts pleiotropic effects on metabolism, satiety, and neural signaling [80,81,82]. Importantly, emerging evidence indicates that subsets of EECs also participate in neurotransmitter signaling, including 5-HT release from enterochromaffin cells and, in some cases, GABA-related signaling pathways, thereby linking endocrine and neurochemical communication within the gut environment. Through both endocrine secretion and paracrine signaling, these cells provide a functional bridge between the intestinal lumen, the ENS, and central neuroendocrine circuits, including the HPA axis.

Activation of the HPA axis, particularly under conditions of physiological or psychological stress, results in the coordinated release of corticotropin-releasing hormone (CRH), adrenocorticotropic hormone (ACTH), and glucocorticoids. These mediators exert profound effects on gut physiology, altering epithelial barrier integrity, mucus production, and immune responses, thereby reshaping the intestinal ecosystem. Clinical evidence supports this connection, demonstrating that acute psychological stress increases small intestinal permeability in humans [83]. Such alterations create a permissive environment for microbial translocation and metabolite flux, amplifying bidirectional signaling along the gut–brain axis. Conversely, the gut microbiome exerts regulatory control over neuroendocrine function. Microbial communities influence the synthesis, metabolism, and availability of key signaling molecules, including 5-HT, cortisol, and their precursors, thereby modulating stress responsiveness and behavioral outcomes. Notably, indigenous spore-forming bacteria stimulate 5-HT biosynthesis by colonic enterochromaffin cells through the production of microbial metabolites that promote host 5-HT synthesis [84], providing a direct mechanism by which microbiota can regulate host serotonergic signaling. Germ-free animal models provide particularly compelling insights: mice devoid of a microbiome exhibit exaggerated HPA axis activation under stress, characterized by elevated CRH, ACTH, and corticosterone levels, alongside marked alterations in anxiety-like behaviors [85]. These findings suggest that microbial colonization is essential for the proper calibration of neuroendocrine stress circuits. The bidirectional nature of this relationship is further underscored by the observation that disruptions in either microbial composition or endocrine signaling can propagate across systems, contributing to disease states. Dysregulation of the HPA axis and associated microbial imbalances have been implicated in a spectrum of CNS disorders [86]. Mechanistically, these conditions are often characterized by a convergence of altered microbial metabolite profiles, immune activation, and neuroendocrine dysfunction, highlighting the interdependence of these pathways.

### 4.4. Barrier Integrity Pathways

Complementing neural and endocrine routes, the regulation of barrier integrity represents a fundamental mechanism through which the gut microbiome influences CNS function. The intestinal barrier serves as a critical interface between the external environment and the host’s internal milieu, maintaining selective permeability while preventing the systemic dissemination of pathogens and harmful metabolites. Structurally, this barrier comprises three principal components: an outer mucus layer, a single layer of epithelial cells interconnected by tight junctions, and an underlying mucosal immune compartment [87]. Transport across this barrier occurs through both transcellular and paracellular mechanisms. Transcellular pathways enable the regulated passage of macromolecules via endocytosis and exocytosis, whereas paracellular transport permits the diffusion of small solutes through intercellular junctions [88]. Disruption of this tightly regulated system results in increased intestinal permeability, facilitating the entry of bacteria and microbial products into the circulation. This phenomenon, often described as “leaky gut,” is associated with systemic inflammation and has been implicated in a range of autoimmune and neurological disorders [86,89].

Microbial metabolism plays a central role in maintaining barrier integrity. Gut bacteria convert dietary substrates, including complex carbohydrates and polyphenols, into bioactive metabolites such as SCFAs, vitamins, and neurotransmitter precursors. These metabolites exert trophic effects on epithelial cells, enhance tight junction protein expression, and modulate immune responses, thereby preserving barrier function. At the same time, they serve as signaling molecules capable of influencing distal organs, including the brain.

The integrity of the BBB represents a parallel and equally critical component of this system. The BBB is a highly specialized structure composed of brain microvascular endothelial cells, pericytes, astrocytes, neurons, microglia, and extracellular matrix components, working in concert to regulate CNS homeostasis [90]. Like the intestinal barrier, the BBB relies on tight junctions to restrict paracellular transport, thereby limiting plasma proteins, pathogens, and neurotoxic substances in the brain while permitting controlled exchange of essential nutrients [91].

Compelling evidence now indicates that gut microbiota and their metabolites can influence BBB integrity. BBB dysfunction has been observed in both animal models of epilepsy and in human epileptic tissue [92], suggesting that barrier disruption may contribute to aberrant neuronal excitability and seizure propagation. Mechanistically, microbial metabolites, including SCFAs such as butyrate and propionate, have been shown to modulate BBB structure and function. Experimental studies demonstrate that these metabolites enhance tight junction integrity and reduce permeability under conditions of stress or injury [93]. For example, administration of *Clostridium butyricum* has been shown to ameliorate BBB impairment in models of traumatic brain injury, likely through increased production of butyrate and associated anti-inflammatory effects [94]. Similarly, propionate has been demonstrated to protect human brain endothelial cells from oxidative stress-induced damage, highlighting a direct mechanism by which microbial metabolites preserve BBB function [95]. Conversely, pathological conditions such as sepsis are associated with increased BBB permeability and enhanced uptake of pro-inflammatory cytokines, including TNF-α, illustrating how systemic inflammation can compromise barrier integrity [96]. Importantly, not all microbial influences are detrimental. Certain bacterial species actively reinforce BBB function by upregulating tight junction proteins. For instance, *Clostridium tyrobutyricum* improves BBB integrity in germ-free mice, an effect attributed to SCFA production, particularly butyrate [97]. Correspondingly, exogenous butyrate administration increases expression of tight junction proteins such as occludin in brain regions including the frontal cortex and hippocampus [98]. The regulation of BBB integrity extends beyond endothelial cells alone, encompassing interactions with pericytes, astrocytes, and microglia, which are cellular components that are themselves responsive to microbial metabolites and systemic inflammatory signals [98,99,100]. This multilayered regulation underscores the complexity of barrier dynamics within the gut–brain axis.

These findings highlight a coordinated system in which intestinal and cerebrovascular barriers function in parallel to maintain physiological homeostasis. Disruptions in microbial composition or metabolite production can destabilize both barriers, propagating inflammatory and neurochemical changes that influence CNS function. Conversely, targeted modulation of the microbiome offers a promising avenue for restoring barrier integrity, thereby providing neuroprotective benefits and potentially mitigating seizure susceptibility.

## 5. Gut-Derived Metabolites Relevant to Epilepsy

The complex interplay between microbial community structure and host physiology is ultimately distilled through a diverse array of bioactive metabolites produced within the gut ecosystem. These microbiota-derived compounds represent a critical functional output of the gut microbiome, translating compositional changes into biologically meaningful signals that can influence local intestinal processes as well as distal organs, including the brain. Disruptions in microbial composition, whether driven by disease, pharmacological interventions, or environmental exposures, can therefore profoundly reshape the metabolic landscape of the gut, altering the spectrum of metabolites that enter systemic circulation and interact with host tissues [101].

A growing body of evidence indicates that many of these metabolites possess neuroactive properties. Some act locally within the gastrointestinal milieu, modulating EEC activity and stimulating the release of hormones and neurotransmitters that feed into gut–brain communication pathways [102]. Others exert endocrine-like effects, traversing the circulation to influence distant targets, including the CNS. Importantly, certain microbial metabolites can modulate BBB integrity or, in some cases, directly cross it to engage neuronal and glial cells, thereby influencing central signaling networks and neuronal excitability [93].

From this perspective, classes of metabolites such as SCFAs, neurotransmitter analogs, and amino acid-derived compounds emerge as key intermediaries linking gut microbial activity to CNS function. These molecules do not act in isolation; rather, they form part of an interconnected signaling network that integrates microbial metabolism with host neurophysiology. As such, understanding their synthesis, transport, and mechanisms of action is essential for elucidating how the microbiome contributes to seizure susceptibility. In the following sections, we focus on neurotransmitter-related metabolites, with particular emphasis on GABA, and highlight emerging candidates such as S-equol that may represent novel therapeutic targets for the modulation of CNS excitability.

### 5.1. Neurotransmitter-Related Metabolites

Among the diverse array of microbiota-derived metabolites, those that interface with classical neurotransmitter systems are of particular relevance to epilepsy. These compounds, which include GABA and other amino acid-derived signaling molecules, directly intersect with pathways governing neuronal excitability, synaptic transmission, and network stability. By influencing the balance between excitatory and inhibitory signaling, they provide a mechanistic link between gut microbial activity and seizure-related processes. In the following section, we review current evidence implicating microbial metabolites in epilepsy and related CNS disorders, with particular emphasis on S-equol as an emerging microbial-derived compound of interest for therapeutic targeting in seizure modulation.

#### 5.1.1. Gamma-Aminobutyric Acid (GABA)

Within the gastrointestinal tract, GABA is produced by multiple microbial taxa, reflecting a conserved metabolic capacity across diverse bacterial lineages. Species within the genera *Bifidobacterium* and *Lactobacillus* have received particular attention due to their abundance of GABA-producing strains and their high efficiency of GABA synthesis [103]. Expanding beyond these well-characterized groups, metabolic profiling, comparative genomics, and phylogenetic analyses have identified GABA production in several Bacteroides strains of human intestinal origin [104]. More broadly, lactic acid bacteria, including *Lactobacillus*, *Bifidobacterium*, and *Streptococcus*, are capable of generating GABA during the fermentation of dietary substrates, highlighting the influence of diet in shaping GABA availability within the gut lumen [105,106,107,108]. At the enzymatic level, microbial GABA synthesis is primarily mediated by glutamate decarboxylase systems. For example, the commensal species *Bifidobacterium dentium* produces GABA through the activity of its GadB enzyme, converting glutamate into GABA as part of its metabolic network [109]. This microbial pathway mirrors the canonical process of GABA biosynthesis in the CNS, where GABA is generated from glutamate by glutamate decarboxylase within inhibitory neurons [110]. Given that GABA is the principal inhibitory neurotransmitter in the brain, perturbations in its synthesis, receptor function, or signaling pathways are closely linked to neuronal hyperexcitability, seizure generation, and epileptogenesis [111]. Conversely, restoration or enhancement of GABAergic signaling is a well-established mechanism underlying many antiseizure therapies.

Accumulating evidence suggests that microbial GABA production can influence central neurophysiology and behavior. Certain bacterial strains, including *Lactobacillus rhamnosus* and *Lactobacillus reuteri*, have been shown to modulate GABAergic signaling pathways through mechanisms that include microbial GABA production, alterations in host GABA receptor expression, and vagus nerve-mediated gut–brain communication. These changes are associated with reductions in anxiety- and depression-related behaviors in experimental models [65]. Diet-induced changes in microbial composition further illustrate this connection; in rats fed a high-fat diet, reductions in prefrontal cortex GABA levels were accompanied by a shift in gut microbial communities, characterized by decreased Bacteroidetes and increased Firmicutes and Cyanobacteria [112]. These observations demonstrate an association between diet-induced alteration in microbial composition and changes in central GABA levels; however, whether the microbiota directly contributes to these neurochemical changes remains unresolved.

Mechanistically, the gastrointestinal tract possesses the molecular infrastructure necessary for GABA-mediated signaling. Both GABA_A_ and GABA_B_ receptors are widely expressed within the enteric nervous system, as well as in epithelial and immune compartments, enabling local modulation of gut physiology and neural signaling [113,114,115,116]. The presence of GABA transporters across the intestinal epithelium further suggests routes by which luminal or microbially derived GABA could enter systemic circulation. Supporting this notion, human studies have demonstrated that interventions altering gut microbiota composition, such as dietary manipulation or fecal microbiota transplantation, can influence systemic GABA levels [47,117,118], while oral GABA administration itself increases circulating concentrations [119].

Experimental models provide additional insight into gut-to-brain GABA signaling. Prebiotic and probiotic interventions have been shown to increase GABA production both in the gut and in brain regions implicated in seizure activity, including the hippocampus and cortex [120]. These effects are thought to arise from enhanced microbial GABA production and indirect modulation of host GABAergic signaling pathways through microbial metabolites and gut–brain communication mechanisms. In germ-free mice, colonization with microbiota derived from individuals with schizophrenia resulted in reduced gut glutamate levels alongside increased hippocampal glutamine and GABA concentrations, coupled with behavioral alterations relevant to neuropsychiatric disease [121]. The use of microbiota transfer into germ-free recipients provides causal evidence that microbial communities can influence GABA-related neurochemistry and behavior. Similarly, administration of *Lactobacillus rhamnosus* (JB-1) in healthy mice induced region-specific changes in GABA_A_ and GABA_B_ receptor subunit expression, decreasing expression in the prefrontal cortex and amygdala while increasing it in the hippocampus, in parallel with reduced anxiety- and depression-related behaviors [65]. Because these effects were observed following experimental manipulation of the microbiome, they provide evidence that specific microbial strains can influence host GABAergic signaling pathways.

Disease models further underscore the functional relevance of microbial modulation of GABA signaling. In an autism spectrum disorder mouse model, reduced abundance of *Lactobacillus reuteri* correlated with diminished expression of GABA receptor subunits in the brain, whereas supplementation restored hippocampal GABA levels and improved cognitive performance [122,123]. Intriguingly, related metabolites such as lactate, whose production is also influenced by microbial activity, have similarly been shown to increase hippocampal GABA levels and enhance memory [123], suggesting that intermediary metabolites may participate in this signaling cascade.

In the context of epilepsy, modulation of the gut microbiota has been directly linked to seizure outcomes through GABA-related mechanisms. Administration of *Lactobacillus*-based probiotics reduced seizure severity in PTZ-kindled rats, potentially via increased brain GABA availability [53], supporting a mechanistic link between gut microbiota and modulation of seizure outcomes. Likewise, administration of the GABA-producing strain *Levilactobacillus brevis* LAB6 causally reduced PTZ-induced seizures, an effect associated with both increased colonic GABA levels and enhanced production of beneficial SCFAs [124]. Furthermore, mono-association of germ-free mice with *Bifidobacterium dentium* demonstrated that this organism alone is sufficient to drive GABA production from multiple substrates, including glutamate, glutamine, and succinate [125]. This finding highlights the metabolic capacity of microbial GABA synthesis and provides direct evidence linking a specific microbial species to host GABA availability. Multiple anatomical and signaling routes may mediate the influence of gut-derived GABA on the CNS. GABA_B_ receptors are expressed along central vagal pathways, particularly within the nucleus tractus solitarius (NTS) and dorsal vagal complex (DVC), providing a plausible neural substrate for microbiota-driven modulation of central GABAergic circuits [126]. Additionally, experimental evidence in rodents suggests that GABA can cross the BBB under certain conditions [107,108,127], although comparable evidence in humans remains limited.

Collectively, these data support a model in which microbial GABA influences CNS function through multiple, potentially convergent mechanisms, including modulation of vagal signaling, alteration of systemic metabolite pools, and regulation of central neurotransmitter systems. However, definitive evidence linking luminal microbial GABA directly to seizure control in humans remains lacking.

#### 5.1.2. Glutamate

In parallel with GABA, glutamate occupies a central position in gut–brain communication, although its biology across the gastrointestinal tract and CNS is governed by markedly different rules of synthesis, utilization, and transport. Within the gut, glutamate arises from multiple sources, including free dietary glutamate and glutamine, the digestion of dietary proteins, endogenous production by gut microbes, and host epithelial metabolism, where it is generated as a by-product of the tricarboxylic acid (TCA) cycle [128]. Yet, despite its abundance in the intestinal lumen, glutamate is not simply a passive nutrient pool. Rather, it is avidly consumed by enterocytes, which preferentially utilize glutamate as a major oxidative substrate, often in preference to glucose, making intestinal glutamate catabolism a key determinant of how much ultimately reaches the portal circulation [129]. This peripheral handling stands in contrast to glutamate metabolism within the CNS, where glutamate must be synthesized locally because circulating glutamate does not readily cross the BBB under normal physiological conditions [130]. Thus, although glutamate is the principal excitatory neurotransmitter in the brain, its central pools are ordinarily insulated from fluctuations in the periphery. This segregation is, however, not absolute. BBB permeability can be altered by stress, dietary factors, inflammation, and microbiome-related changes, with experimental evidence suggesting that such perturbations may permit increased glutamate accumulation in both blood and brain [131]. These observations raise the possibility that gut-derived glutamate, even if indirectly, may contribute to central excitatory tone under conditions of barrier compromise.

Several mechanisms may link luminal glutamate to the CNS. Glutamate can be transported across the intestinal barrier and detected by vagal afferent endings innervating the gastrointestinal tract, or it can be absorbed through glutamate transporters expressed on the apical membrane of intestinal epithelial cells [131]. Supporting evidence from infant pigs demonstrates that glutamate absorption and transport into the portal circulation increase with higher intraduodenal glutamate intake, although only a small fraction escapes intestinal utilization and systemic glutamate and brain glutamate concentrations remain largely unchanged [132]. This study demonstrates that luminal glutamate availability can influence intestinal absorption and portal glutamate transport, although it does not establish a corresponding increase in brain glutamate levels. Consistent with this, glutamate transport systems are prominently expressed on the apical surface of epithelial cells in the small intestine and, to a lesser extent, in the stomach, thereby providing the molecular machinery required for luminal sensing and regulated uptake [133].

The gastrointestinal tract is also equipped with a robust receptor system for glutamatergic signaling. Ionotropic and metabotropic glutamate receptors (mGluRs) have been identified on epithelial cells and enteric neurons throughout the stomach, small intestine and colon [134,135,136,137,138,139,140]. This broad receptor distribution indicates that glutamate may function locally as a signaling molecule in addition to serving as a metabolic substrate. Indeed, glutamate can engage gut–brain communication indirectly through vagal pathways; luminal administration of glutamate to the rat stomach increases vagal afferent firing and activates downstream gut–brain signaling circuits [141]. These findings suggest that, even in the absence of substantial systemic transport, luminal glutamate can be transduced into neural signals capable of influencing central processing.

Microbial communities shape both fecal and plasma glutamate profiles and shifts in microbial composition can alter the balance of glutamate synthesis, consumption, and downstream conversion to related metabolites such as GABA and glutamine [142]. Such changes may have functional consequences for brain excitability. For example, glutamate dysregulation has been implicated in schizophrenia, and germ-free mice colonized with microbiota associated with schizophrenia-related phenotypes exhibited lower gut glutamate levels, reduced microbial α-diversity, and elevated hippocampal glutamine and GABA [121]. These findings support the concept that microbial remodeling of peripheral glutamate metabolism can reverberate through central amino acid and neurotransmitter pathways.

A similar convergence of immune and excitatory mechanisms is evident in depression. Individuals with depression exhibit elevated circulating levels of proinflammatory cytokines such as TNF-α and IL-6 compared with healthy controls [143], suggesting that peripheral inflammatory signaling contributes to central dysfunction. These cytokines, in turn, modulate glutamatergic neurotransmission by increasing synaptic glutamate release, enhancing glutamate receptor activity and impairing astrocytic glutamate clearance [144]. Collectively, these effects elevate excitatory signaling and promote excitotoxic pathways linked to neurodegeneration. This provides an indirect and biologically plausible route through which peripheral inflammatory and metabolic disturbances, including those shaped by the gut microbiome, may intersect with glutamatergic signaling to influence brain function.

Although dietary intake contributes substantially to luminal glutamate, the abundance of glutamate within the gut is also strongly shaped by microbial ecology. Certain bacterial families show positive associations with fecal or circulating glutamate levels, whereas others are inversely correlated, underscoring the importance of microbial composition in determining glutamate bioavailability [142]. In a human study, the relative abundance of *Coriobacteriaceae* was positively associated with plasma glutamate, whereas *Streptococcaceae* showed a negative correlation with fecal glutamate levels [142]. These associations suggest that microbial community structure may contribute to variation in glutamate availability, although causal relationships between specific taxa and host glutamate pools remain to be established.

These relationships may be particularly relevant in epilepsy. Comparative analyses of the gut microbiota of patients with epilepsy and healthy family members revealed an increased abundance of *Verrucomicrobia* in epilepsy, accompanied by a corresponding increase in glutamate levels [145]. This finding represents an association between epilepsy status, microbial composition and glutamate levels rather than direct evidence that microbial glutamate contributes to seizure generation. Nevertheless, it raises the possibility that microbial shifts favoring excitatory metabolite profiles may contribute to seizure susceptibility. This interpretation is strengthened by evidence from dietary interventions known to modify both microbial composition and seizure outcomes. The KD, a well-established therapy for drug-resistant epilepsy, induces characteristic restructuring of the gut microbiome [45,49]. In rodent seizure models, microbiota-dependent experiments demonstrated that ketogenic diet-induced enrichment of *Akkermansia* and *Parabacteroides* was necessary and sufficient for seizure protection and was accompanied by an increased hippocampal GABA/glutamate ratio [21], suggesting that microbiome-mediated modulation of excitatory and inhibitory neurotransmission contributes to the antiseizure effects of the ketogenic diet. Thus, although ketogenic therapy is often framed in metabolic terms, its effects may also depend in part on microbiome-mediated recalibration of excitatory–inhibitory neurotransmitter balance.

Further support for this concept comes from studies of glutamate-metabolizing bacteria. Oral administration of *Bifidobacterium adolescentis* IPLA60004, a strain capable of converting glutamate to GABA, reduced circulating glutamate concentrations [146], demonstrating that targeted bacterial metabolism can alter peripheral glutamate pools. Such findings suggest that targeted manipulation of microbial glutamate metabolism may represent a plausible strategy for attenuating peripheral excitatory load and, potentially, downstream neuronal hyperexcitability. Yet, important caveats remain. Glutamate itself is not readily permeable to the BBB under physiological conditions, and systemic transport is additionally constrained by competing metabolic demands, including glutathione synthesis, which consumes glutamate within the gut and other peripheral tissues [133]. These factors complicate any direct causal link between luminal glutamate and central glutamatergic transmission.

The available evidence suggests that gut-derived glutamate may influence CNS function through several convergent mechanisms: local sensing by epithelial and enteric glutamate receptors, activation of vagal afferent pathways, microbiome-dependent modulation of glutamate and related amino acid pools, and indirect effects on inflammatory and excitatory signaling networks. However, as with microbial GABA, the extent to which gut glutamate directly contributes to seizure susceptibility remains unresolved. More targeted studies are therefore needed to clarify how intestinal glutamate synthesis, uptake, microbial metabolism, and neural sensing intersect with central excitability and epileptogenesis.

#### 5.1.3. Tryptophan/Serotonin

In parallel with GABA and glutamate, tryptophan and its downstream serotonergic metabolites occupy a central position in gut–brain communication. Yet they do so through a distinct metabolic logic in which precursor availability, compartment-specific synthesis, and receptor-level signaling all shape neurophysiological outcomes. Tryptophan is an essential amino acid that cannot be synthesized endogenously and must therefore be obtained through dietary protein [147]. Once absorbed in the gut, tryptophan enters the circulation and can cross the BBB through a specialized amino acid transporter, thereby supplying substrate for 5-HT synthesis within the CNS [147]. By contrast, 5-HT itself does not readily cross the BBB [148], necessitating independent serotonergic pools in the periphery and brain. In the periphery, the vast majority of 5-HT is produced within the gastrointestinal tract, where enterochromaffin cells and enteric neurons synthesize it from tryptophan using the rate-limiting enzyme tryptophan hydroxylase 1 (TPH1) [149,150,151]. This peripheral serotonergic system exerts broad effects on gastrointestinal physiology through a diverse repertoire of receptor subtypes, most of which are G protein-coupled receptors expressed across the gut epithelium, enterochromaffin cells, and enteric neurons [152,153,154]. Through these receptors, 5-HT regulates peristalsis, secretion, vasodilation, nociception, and nausea, underscoring its central role in coordinating intestinal homeostasis [152,153,154]. Peripheral serotonergic signals also communicate with the CNS through vagal pathways. Consistent with this, vagus nerve stimulation, an established therapy for drug-resistant epilepsy, has been shown to enhance serotonergic signaling, including significant increases in extracellular 5-HT in experimental models [155].

Within the CNS, 5-HT is synthesized locally from L-tryptophan by the neuron-specific enzyme TPH2. Central 5-HT influences mood, appetite, memory, stress responsiveness, and pain perception [153,154]. Its effects on neuronal excitability are complex and highly context-dependent. Depending on receptor subtype, cellular localization, and circuit state, serotonergic signaling can promote either depolarization or hyperpolarization by modulating both glutamatergic and GABAergic transmission [156,157,158]. Thus, rather than acting as a uniformly inhibitory or excitatory signal, 5-HT is better understood as a dynamic regulator of network tone, capable of shifting the balance of excitation and inhibition in ways that may either protect against or facilitate seizure generation [156,157,158].

Experimental and associative evidence indicates that the gut microbiome shapes the serotonergic system, beginning early in development. The maturation of gut microbial communities overlaps with the development of the enteric serotonergic network, in part because gut microbes regulate intestinal 5-HT production and signaling during a critical developmental window [150]. This temporal relationship supports an association between microbial colonization and serotonergic maturation, although colonization studies are needed to establish causality for specific developmental effects. Microbiota-mediated increases in enterochromaffin-derived 5-HT promote enteric neuron proliferation and maturation through serotonergic pathways, thereby linking microbial colonization to the developmental assembly of gut neural circuits [159]. This relationship is highlighted in germ-free mice, which exhibit pronounced abnormalities in 5-HT metabolism, including reduced intestinal 5-HT levels [160] and decreased colonic TPH1 expression; notably, these deficits can be normalized by colonization with conventional or human microbiota [161]. Restoration of intestinal 5-HT levels and TPH1 expression following microbiota colonization provides evidence of a direct role for microbial regulation of intestinal serotonergic pathways.

Specific microbial strains have been shown to increase enteric 5-HT availability through distinct metabolic mechanisms. Administration of Escherichia coli Nissle 1917 increased levels of 5-hydroxytryptophan and reduced its breakdown product, 5-hydroxyindoleacetic acid (5-HIAA) [162], supporting a direct effect of this strain on host serotonergic profile. Colonization with *Clostridium ramosum* in germ-free mice likewise increases host 5-HT levels [159], showing that a defined bacterial species can modulate host serotonergic metabolism. In addition, multiple lactic acid bacteria, including *Bifidobacterium dentium*, *B. longum*, *Bifidobacterium infantis*, *Lacticaseibacillus rhamnosus*, *Lactobacillus acidophilus*, and *Limosilactobacillus reuteri*, have been implicated in the regulation of tryptophan and 5-HT metabolism [163]. For example, probiotic administration of *Bifidobacterium infantis* has been associated with increased circulating tryptophan and reduced 5-HIAA, consistent with altered 5-HT turnover [164]. Beyond effects on 5-HT synthesis itself, microbial metabolites such as SCFAs can modulate serotonergic signaling by regulating 5-HT transporter function and altering the expression of multiple 5-HT receptor subtypes. In cell culture models, individually and combined SCFAs modulated 5-HT transporter function and altered expression of several 5-HT receptor subtypes including 5-HT1A, 5-HT2B, and 5-HT7 [165], indicating a mechanistic role for microbial metabolites in regulating components of serotonergic signaling. These findings suggest that the microbiome influences serotonergic tone not only by regulating precursor availability and synthesis but also by shaping receptor responsiveness.

Evidence linking serotonergic signaling to epilepsy further underscores the relevance of this pathway to seizure susceptibility. Elevations in extracellular 5-HT suppress both focal and generalized seizures, whereas depletion of brain 5-HT lowers seizure threshold and increases susceptibility to convulsions [166,167,168]. In the KA model of epilepsy, depletion of 5-HT increased tonic–clonic seizure susceptibility and lethality [169], providing causal evidence that reduced central serotonergic tone can worsen seizure outcomes. Conversely, activation of the 5-HT1A receptor, which hyperpolarizes hippocampal neurons, has been associated with increased seizure threshold [170]. At the same time, other studies have reported proconvulsant actions of 5-HT1A and additional serotonergic receptor subtypes, emphasizing that the net effect of 5-HT depends on receptor distribution, circuit context, and the broader neurochemical environment [171,172]. This context dependency is further highlighted by the fact that excessive serotonergic activity can itself be harmful; severe 5-HT syndrome is characterized by hyperthermia and seizures, demonstrating that both serotonergic deficiency and excess may destabilize neural networks [173].

Tryptophan metabolism through the kynurenine pathway also appears to be relevant to epilepsy. In a human study of patients with refractory epilepsy undergoing KD therapy, specific blood kynurenine metabolites were associated with therapeutic response [174], suggesting a potential link between tryptophan catabolism and seizure control, but not establishing causality. Strong mechanistic support for this concept comes from infantile spasms, where inhibition of the indoleamine 2,3-dioxygenase 1–kynurenine pathway enhances production of the anticonvulsant metabolite kynurenic acid and reduces spasms [175]. These findings extend the significance of tryptophan metabolism beyond 5-HT alone and suggest that microbiome-sensitive tryptophan flux may influence seizure susceptibility through multiple downstream branches.

Microbiome-associated serotonergic mechanisms are also evident in epilepsy-related models and patient studies. A pediatric epilepsy plasma metabolomics study reported reduced tryptophan and 5-HIAA levels relative to age-matched controls, providing associative evidence of altered serotonergic metabolism in the disease state [176]. However, these findings do not establish whether altered tryptophan/5-HT metabolism contributes to seizure susceptibility or reflects downstream consequences of epilepsy or related metabolic changes. Altered serotonergic signaling has likewise been implicated in sudden unexpected death in epilepsy (SUDEP), where dietary modulation of tryptophan confers protective effects. In the DBA/1 mouse model of SUDEP, a high-tryptophan diet increased brain 5-HT levels and reduced seizure activity, providing evidence that dietary tryptophan can modulate seizure outcomes [58]. These effects were accompanied by shifts in gut microbial composition, including increased Proteobacteria and Actinobacteria [58], although the direct role of these microbial changes was not established. In a related DBA/1 study, seizure-induced respiratory arrest significantly decreased levels of the 5-HT precursor 5-hydroxytryptophan (5-HTP), whereas exogenous administration of 5-HTP reduced seizure-induced respiratory arrest [177]. This intervention demonstrates that serotonergic precursor availability can modulate this seizure-related phenotype.

Although 5-HT itself does not cross the BBB, several mechanisms may nevertheless permit gut-derived or microbiome-modulated serotonergic signaling to influence brain excitability. One plausible route is through vagal pathways, which are essential for certain antidepressant effects that depend on serotonergic signaling [178,179,180]. This provides a mechanistic basis through which peripheral serotonergic changes, even without direct BBB passage, may alter central excitability. Consistent with this view, some antidepressants that enhance serotonergic tone have shown promise as adjunctive antiseizure therapies [181].

Together, these observations support a functional link between tryptophan availability, serotonergic signaling, microbial composition and seizure-associated outcomes. The gut microbiota can influence tryptophan availability, intestinal 5-HT synthesis, receptor signaling, and downstream kynurenine metabolism, each of which has the potential to shape neuronal excitability and seizure susceptibility. However, as with other microbiota-derived neurotransmitter-related pathways, direct evidence that gut-derived 5-HT itself modulates seizure activity in humans remains limited. Further studies are therefore needed to define how microbial regulation of tryptophan and 5HT metabolism intersects with neural circuits involved in epileptogenesis and seizure control.

#### 5.1.4. Dopamine

Extending beyond amino acid-derived inhibitory and excitatory systems, dopaminergic signaling represents an additional layer through which the gut microbiome can interface with host neurophysiology. As with the serotonergic system, dopamine biology within the gut–brain axis is defined by compartmentalized synthesis, localized signaling, and indirect pathways of communication with the CNS. Within the gastrointestinal tract, dopamine is synthesized through a two-step enzymatic process in which tyrosine is converted to L-3,4-dihydroxyphenylalanine by tyrosine hydroxylase, followed by decarboxylation to dopamine [182]. This synthesis is not restricted to neurons; rather, it occurs across a range of peripheral cell types, including epithelial cells, enteric neurons, immune cells, and, notably, select microbial populations [183].

Remarkably, the gut represents a major source of peripheral dopamine, with estimates indicating that more than half of total body dopamine is produced outside the CNS, resulting in concentrations that exceed those found centrally [184,185]. In contrast to classical neurotransmission within the brain, peripheral dopamine largely acts in an autocrine and paracrine manner, binding to dopamine receptors on neighboring cells to regulate gastrointestinal motility, maintain barrier integrity, and modulate inflammatory responses [184,186]. These functions position dopamine as a key regulator of intestinal homeostasis, analogous to the roles described for GABA and 5-HT in coordinating gut physiology.

Despite this substantial peripheral pool, circulating dopamine does not readily cross the BBB and is rapidly degraded by monoamine oxidase and catechol-O-methyltransferase into inactive metabolites [187]. Consequently, as with peripheral 5-HT, dopamine must be synthesized locally within the CNS to influence neural circuit activity. Several microbial taxa contribute to the dopaminergic milieu within the gut. Dopamine-producing species include *Bacillus cereus*, *Bacillus mycoides*, *Bacillus subtilis*, *Proteus vulgaris*, *Serratia marcescens*, *Staphylococcus aureus*, *Escherichia coli*, *Hafina alvei*, and *Klebsiella pneumoniae* [188,189]. In addition, broader microbial communities including *Prevotella*, *Bacteroides*, *Lactobacillus*, *Bifidobacterium*, *Clostridium*, *Enterococcus*, and *Ruminococcus* have been implicated in modulating dopaminergic pathways, highlighting the distributed nature of microbial influence on this system [190,191].

Microbial metabolites further shape dopaminergic signaling by regulating key biosynthetic enzymes. For example, in vitro studies demonstrated that butyrate upregulates tyrosine hydroxylase expression in PC12 cells [192], indicating a mechanistic role for microbial metabolites in regulating dopamine biosynthesis. Such findings suggest that microbial metabolic activity can influence dopaminergic tone not only through direct neurotransmitter production but also by controlling precursor availability and enzymatic capacity within host cells.

Within the CNS, dopamine is synthesized in discrete brain regions, including the substantia nigra, ventral tegmental area, and hypothalamus, where it exerts widespread regulatory effects on motor control, reward processing, motivation, and cognitive function [193,194]. Dopaminergic signaling operates through G protein-coupled receptors to regulate ion channel activity, thereby influencing neuronal excitability at both synaptic and circuit levels [194]. Unlike GABA, which predominantly suppresses excitability, or glutamate, which promotes it, dopamine functions as a modulatory transmitter whose effects depend on receptor subtype and cellular context. This receptor-specific complexity is particularly evident in epilepsy. Activation of D1-like receptors generally promotes neuronal excitability, whereas D2-like receptor signaling often exerts anticonvulsant effects, resulting in bidirectional and sometimes opposing influences on seizure susceptibility [195]. In line with these findings, altered expression of dopamine receptors and transporters, as well as changes in dopamine availability, have been reported in patients with epilepsy [196,197,198,199]. Furthermore, both experimental and clinical studies support a role for dopaminergic circuits in epileptogenesis and seizure propagation, although the precise mechanisms remain incompletely defined [199,200].

Although dopamine does not cross the BBB, its precursor L-tyrosine does, providing a mechanistic route through which peripheral metabolic changes may influence central dopamine synthesis [201]. Systemically, L-tyrosine is derived from dietary phenylalanine via phenylalanine hydroxylase in the liver before entering the brain to support dopaminergic neurotransmission [202]. This dependence on precursor availability introduces a potential interface through which diet and microbiome-dependent amino acid metabolism may indirectly modulate CNS dopamine levels. Dopamine receptors are expressed throughout the gastrointestinal tract, including the ileum, colon, stomach, duodenum, and esophagus, highlighting the relevance of dopaminergic signaling in peripheral tissues [203,204,205,206,207,208,209,210,211,212,213,214]. In the liver, phenylalanine hydroxylase converts L-phenylalanine to L-tyrosine, which subsequently crosses the BBB to support dopamine synthesis within the brain [202].

The microbiome sensitivity of dopaminergic pathways is further supported by germ-free animal models. Germ-free mice exhibit alterations in tyrosine hydroxylase expression, region-specific changes in brain dopamine concentrations, and reduced peripheral tyrosine hydroxylase mRNA levels, indicating that microbial signals are required for normal dopaminergic system development and function [215]. Consistent with this, mono-association of germ-free mice with *Bifidobacterium dentium* increases both fecal and brain tyrosine levels [125], supporting a role for a single microbial species in modulating dopamine precursor availability.

Emerging evidence suggests that microbiome-derived modulation of dopamine pathways can influence CNS function and behavior more broadly. A large-scale metagenomic study in healthy individuals found that increased microbial production of dopamine-related metabolites was associated with improved mental well-being, with butyrate-producing bacteria such as *Faecalibacterium* and *Coprococcus* positively associated with quality-of-life measures [216]. However, the causal relationship between metabolites associated with dopamine and psychological outcomes was not evaluated. In neurodegenerative disease, particularly Parkinson’s disease, a prevailing hypothesis posits that pathology may originate within the gut or peripheral nervous system before propagating to the brain [217], implicating microbiome and dopamine interactions in disease progression, although direct evidence remains incomplete. Supporting this concept, probiotic administration of *Lactobacillus plantarum* PS128 improves motor symptoms in Parkinsonian mouse models, suggesting functional restoration of dopaminergic signaling pathways [218]. This intervention provides causal evidence that microbiome manipulation can influence dopamine-related neurological outcomes in vivo.

Additional insights come from neurodevelopmental and neuropsychiatric models. In a rodent autism model, deletion of the cell adhesion gene *EphB6* resulted in gut microbiota dysbiosis, vitamin B6 deficiency, altered dopamine signaling, and behavioral abnormalities [219]. In the same study, fecal microbiota transplantation experiments demonstrated that gut microbiota-dependent alterations in vitamin B6 and dopamine metabolism contribute to changes in behavior and cortical excitation/inhibition balance. While direct evidence connecting peripheral dopaminergic alterations to epilepsy is more limited, emerging studies suggest potential associations. For example, increased dopamine receptor expression has been observed in peripheral immune cells from patients with temporal lobe epilepsy [220], and elevated fecal dopamine levels have been reported in children with cerebral palsy and comorbid epilepsy compared to non-epileptic controls [221].

Collectively, these observations suggest a model in which microbiome-dependent modulation of dopaminergic pathways may influence CNS excitability through indirect mechanisms, including regulation of precursor availability, enzyme expression, and peripheral signaling systems. However, the current data remains largely associative and does not provide strong direct evidence for a causal link between gut-derived dopamine and epileptogenesis, and the underlying mechanisms remain incompletely defined.

### 5.2. Short-Chain Fatty Acids (SCFAs)

Among the gut-derived metabolites implicated in host neurophysiology, SCFAs occupy a particularly important position because they couple microbial fermentation to immune, metabolic, and neural signaling across the gut–brain axis. SCFAs are saturated fatty acids containing one to six carbon atoms [23], generated predominantly in the colon through the microbial fermentation of indigestible dietary fibers that escape digestion in the small intestine [222,223]. In this way, SCFAs may be viewed not merely as metabolic end products, but as bioactive intermediaries through which the gut microbiota exerts distal effects on host tissues, including the CNS.

Mechanistically, SCFAs exert their effects through both receptor-dependent and receptor-independent pathways. They serve as endogenous ligands for several G protein-coupled receptors, including FFAR2, FFAR3, hydroxycarboxylic acid receptor 1 (GPR109A/HCAR2), and GPR164 [224]. These receptors are expressed on ECCs, immune cells, and other peripheral tissues, where they regulate energy homeostasis, inflammatory tone and gut–immune communication [225,226]. Although their roles within the nervous system remain less fully resolved, SCFAs have been implicated in biological processes highly relevant to brain function, including cognition, energy balance, chronic pain, neuroinflammation, and the secretion of neuroactive gut peptides such as peptide YY, insulin and glucagon-like peptide-1 [227]. Beyond these systemic effects, in vitro evidence indicates that butyrate can directly modulate hippocampal synaptic plasticity by enhancing long-term potentiation [228], suggesting that SCFAs may influence neuronal excitability not only indirectly through peripheral signaling, but also through more proximal effects on neural function.

The capacity of SCFAs to influence CNS physiology depends, in part, on their ability to move beyond the gut lumen and engage barriers, immune, and metabolic pathways that shape the brain’s microenvironment. Experimental studies in both rodents and humans demonstrate that SCFAs, particularly acetate, can cross the BBB via monocarboxylate transporters [229,230,231]. Once available to the CNS, SCFAs participate in several mechanisms relevant to seizure biology. They help maintain BBB integrity by strengthening tight junctions and reducing permeability under inflammatory conditions [95,98,232], an effect of particular importance given the growing evidence that barrier dysfunction contributes to epileptogenesis. SCFAs can also influence neurotransmitter systems linked to excitability. Acetate participates in metabolic pathways that support GABA synthesis [233], while acetate and butyrate have both been shown to increase 5-HT levels in chromaffin cells [84]. These observations place SCFAs at the intersection of barrier regulation, neuroimmune signaling, and neurotransmitter metabolism, thereby providing several plausible routes through which microbial fermentation products could influence seizure susceptibility.

A growing body of evidence now implicates altered SCFA signaling in epilepsy. Clinical studies have reported associations between disrupted SCFA profiles and several neurological conditions, including epilepsy [234]. In individuals with epilepsy, these changes include alterations in both circulating and fecal SCFA levels, together with disruptions in butyrate-producing microbial taxa [235,236]. Patients with drug-resistant epilepsy have been reported to exhibit reductions in several SCFA-producing taxa [32]; however, these studies do not establish whether SCFA alterations contribute to disease severity or arise as a consequence of epilepsy or treatment. A study in children with intractable epilepsy found decreased abundance of butyrate-producing bacteria that correlated with both seizure severity and responsiveness to the KD [237], providing associative evidence linking SCFA-producing taxa to disease outcomes. These observations suggest that SCFA disruption may be functionally linked to disease severity and treatment response; however, the causal nature of these relationships remains unclear.

Preclinical findings provide further mechanistic support for such a relationship. In germ-free mice, the absence of the microbiota leads to dysregulated expression of genes involved in controlling excitatory/inhibitory signaling, an effect that can be reversed by colonization with specific-pathogen-free microbiota [234], supporting a direct role for the microbiota in regulating these pathways. Given that SCFAs influence glutamate, glutamine, and GABA metabolism [233], they represent a biologically plausible intermediary through which microbial composition could shape neuronal excitability. Consistent with this, administration of *Lactobacillus helveticus* R0052 increased acetate and butyrate levels while simultaneously raising seizure thresholds in the 6-Hz seizure test [238]. These findings suggest that microbiome-dependent SCFA production can influence seizure susceptibility. Similarly, intragastric administration of butyrate and propionate prolonged latency to PTZ-induced seizures and reduced Racine seizure scores [239]. Taken together, these findings support the concept that SCFAs can dampen neuronal hyperexcitability and mitigate seizure severity, although the relative contributions of individual SCFAs, receptor pathways, and downstream metabolic changes remain to be fully clarified.

The relationship between SCFAs and established antiseizure therapies adds a further layer of complexity. At present, interactions between SCFAs and ASMs remain incompletely characterized, reflecting a broader gap in understanding how microbiome-derived metabolites influence pharmacological responsiveness. Valproic acid, one of the most widely used ASMs, has been reported to alter SCFA production in the marine fungus *Trichoderma reesei*, where it both inhibited and induced specific circulating SCFAs [240]. However, comparable findings in humans have not been consistent. In one study, addition of valproic acid in patients with epilepsy did not significantly alter plasma or urinary SCFA concentrations [241]. By contrast, changes in SCFA profiles have been reported in individuals with epilepsy undergoing KD therapy [242], raising the possibility that dietary interventions may more robustly reshape SCFA-related pathways than conventional pharmacotherapy alone. These divergent findings underscore the need to distinguish drug-specific effects from broader metabolic and microbiome-mediated adaptations.

Taken together, the available evidence positions SCFAs as compelling molecular intermediaries linking gut microbial fermentation to seizure-relevant pathways in the host. Through their ability to engage G protein-coupled receptors, preserve barrier integrity, modulate immune signaling, influence neurotransmitter metabolism, and, in some cases, access the CNS directly, SCFAs provide a mechanistic framework through which the microbiome may influence epileptogenesis and seizure control. Yet, despite this promise, their therapeutic potential remains incompletely defined. Future work will need to determine how specific SCFAs move from the gut lumen into systemic and cerebral compartments, which cellular targets they engage once there, and how their actions integrate with diet, microbiota composition, and existing antiseizure therapies to shape seizure susceptibility and outcome.

### 5.3. Energy-Related Metabolite—Lactate

In parallel with SCFAs and neurotransmitter-related metabolites, lactate is increasingly recognized as an important intermediary linking microbial metabolism to host neurophysiology. Once regarded primarily as a by-product of anaerobic metabolism, lactate is now understood to function both as an energy substrate and as a signaling molecule, thereby expanding its relevance well beyond cellular bioenergetics. Within the gastrointestinal tract, lactate is generated through anaerobic fermentation by a wide range of microbial taxa, including members of the Firmicutes, Proteobacteria, Bacteroidetes, and Actinobacteria phyla, with many *Bifidobacterium* species serving as prominent producers [243,244,245]. In microbial communities, lactate production contributes to redox balance, yet its accumulation is not uniformly beneficial. Excess luminal lactate is associated with reduced pH and adverse outcomes such as lactic acidosis and colitis [246,247,248]. Under homeostatic conditions, however, lactate is further metabolized by lactate-utilizing bacteria into the beneficial SCFAs butyrate and propionate, thereby preventing its accumulation and integrating lactate metabolism into broader trophic networks that support intestinal health [249,250].

Within the CNS, lactate arises from several sources, including astrocytic glycolysis of blood-derived glucose, circulating lactate, and glycogen stores, and it serves as a major substrate for neuronal energy metabolism [251]. This view, however, captures only part of its biological significance. Lactate is now established as an active signaling molecule capable of mediating intercellular communication across multiple tissues, including skeletal muscle and brain [251]. In the nervous system, astrocyte-derived lactate has been implicated in memory consolidation, neuronal signaling, and neuroprotection [244,252,253,254], underscoring its importance in sustaining activity-dependent brain function. Thus, as with other metabolites discussed in the gut–brain–microbiome axis, lactate occupies a functional space in which metabolism and signaling converge.

The movement and action of lactate within the CNS are tightly regulated. Because lactate does not freely diffuse across the BBB, its transport depends on monocarboxylate transporters (MCTs), after which it can be enzymatically converted to pyruvate within neurons and glia [255]. Beyond this metabolic role, lactate can also exert receptor-mediated effects through HCAR1/GPR81, a G protein-coupled receptor expressed in peripheral tissues such as muscle and adipose tissue, as well as in the brain [256]. In the CNS, HCAR1 is localized to hippocampal pyramidal neurons, interneurons, Purkinje cells, and the postsynaptic membrane of excitatory neurons [256,257]. Activation of this receptor influences inflammatory signaling, cell survival, and neuronal excitability through intracellular pathways involving alpha and beta gamma subunits of inhibitory G protein (Giα and Giβγ), and Ras/Raf/Mitogen-activated protein kinase/ERK kinase (MEK)/extracellular-signal-regulated kinase (ERK) [258]. These observations place lactate in a position of mechanistic interest, as it may influence neural function through both substrate delivery and dedicated signaling pathways.

The gut microbiome is positioned to shape this system in several ways. At the most immediate level, microbial communities determine the extent of luminal lactate production and its subsequent conversion into other metabolites, thereby influencing whether lactate accumulates or is efficiently funneled into beneficial downstream pathways. Because lactate-producing and lactate-consuming bacteria jointly determine intestinal lactate availability, shifts in community composition could alter the amount of lactate entering circulation and potentially the metabolic signals reaching the CNS. Although the movement of gut-derived lactate from the intestinal lumen to the brain remains incompletely mapped, the existence of MCT-mediated transport and receptor-based sensing provides a plausible mechanistic basis through which peripheral lactate could influence central function [255,256]. In this respect, lactate resembles other microbiome-sensitive metabolites that may act locally in the gut while also participating in more distal signaling networks.

The relevance of lactate to epilepsy emerges most clearly when metabolic stress and excitability are considered together. Seizures impose marked energetic demands on neural tissue, and lactate accumulation is consistently observed during severe seizure activity [259], although this observation alone does not establish whether elevated lactate contributes to seizure generation or simply reflects the metabolic demands of seizures. However, experimental evidence indicates that lactate is not merely a passive consequence of this metabolic strain, but may contribute to epileptic activity itself. Across multiple seizure models, inhibition of lactate dehydrogenase (LDH), the enzyme responsible for L-lactate production, reduces seizure activity [260,261], supporting a mechanistic role for lactate metabolism in sustaining neuronal hyperexcitable states. In this context, altered lactate handling may represent one component of the broader metabolic reprogramming that accompanies epileptogenesis.

Microbiome-related and dietary influences on lactate pathways may also intersect with seizure susceptibility. In a PTZ seizure model, increased lactate production enhanced GABA metabolism and contributed to the antiseizure effects of the KD [54], implicating lactate metabolism in seizure modulation. This observation is particularly notable because it places lactate within an established therapeutic setting for epilepsy and suggests that its actions may extend beyond energy provision to influence inhibitory neurotransmitter systems. Additional mechanistic support comes from a recent study showing that conditional knockout of *LDHA*, the gene encoding LDH-A, reduced L-lactate levels and decreased neuronal excitability in the dorsomedial prefrontal cortex, whereas *LDHA* overexpression exerted the opposite effect [262], demonstrating that manipulation of lactate metabolism directly alters neuronal excitability. Together, these findings support a model in which lactate availability is linked to neuronal excitability in a bidirectional and context-dependent manner.

Lactate may also interact with seizure-relevant pathways through effects on neuroplasticity. Brain-derived neurotrophic factor (BDNF), a key regulator of synaptic plasticity and neuronal excitability, exhibits bidirectional changes in epilepsy [263]. Peripheral lactate administration has been shown to increase central BDNF levels and enhance neuroplasticity [264], raising the possibility that systemic or microbiome-influenced lactate can directly affect seizure-related processes through trophic signaling pathways in addition to its metabolic and receptor-mediated actions. While these findings do not yet establish a direct causal role for gut-derived lactate in epileptogenesis, they further broaden the mechanistic landscape through which lactate may influence seizure biology.

Altogether, the available evidence positions lactate as a multifaceted metabolite at the intersection of microbial fermentation, host metabolism, and neuronal signaling. It functions not only as an energy substrate but also as a signaling molecule capable of influencing inflammatory pathways, neuroplasticity and neuronal excitability. Yet, despite these advances, the contribution of gut-derived lactate, and of the microbes that produce or consume it, to CNS function and epileptogenesis remains incompletely defined.

### 5.4. Microbiota-Dependent Vitamins

Extending beyond metabolites derived from microbial fermentation and amino acid metabolism, vitamins represent a class of microbiota-influenced compounds that bridge nutrient availability with systemic physiological regulation. Traditionally defined as essential micronutrients required for normal growth, development, and cellular function, vitamins are obtained primarily through dietary intake, as mammals lack the capacity to synthesize most of these compounds endogenously. However, a substantial body of evidence now indicates that the gut microbiota contributes meaningfully to the production and bioavailability of several vitamin classes, thereby integrating microbial metabolic activity into host nutritional and metabolic networks.

Within this framework, vitamins should be viewed not solely as passive cofactors but as dynamic participants in the broader signaling landscape of the gut–brain axis. In addition to their well-established roles in energy metabolism, particularly in facilitating the conversion of macronutrients into adenosine triphosphate, many vitamins modulate immune responses, inflammatory pathways, oxidative stress, and neurotransmitter synthesis. These processes intersect directly with mechanisms implicated in epileptogenesis. Accordingly, alterations in vitamin availability may influence seizure outcomes through both direct and indirect mechanisms, shaping neuronal excitability and circuit stability while also modifying systemic factors that contribute to disease progression.

In the sections that follow, we synthesize current evidence linking vitamin metabolism to microbiome function and CNS regulation, with a particular emphasis on epilepsy. We focus on vitamins that are either produced or modulated by the gut microbiota and that have demonstrated mechanistic relevance to neuronal excitability, neurotransmitter metabolism, inflammation, or oxidative stress. Through this lens, we consider how dietary modulation, microbial composition, and targeted supplementation strategies may be leveraged to influence these pathways, with the goal of identifying adjunctive approaches to seizure management grounded in the biology of the gut–brain–microbiome axis.

#### 5.4.1. Folate (Vitamin B9)

Among microbiota-dependent vitamins, folate represents one of the most extensively characterized links between microbial metabolism, host physiology and CNS function. As an essential micronutrient, folate plays a central role in one-carbon metabolism, supporting (deoxyribonucleic acid) DNA synthesis, methylation reactions, and numerous aspects of cellular homeostasis [265]. These biochemical functions position folate at a critical intersection between metabolism and signaling, where subtle perturbations can propagate across multiple physiological systems, including those governing neuronal integrity and excitability.

Although dietary intake remains the primary source of folate, the gut microbiota contributes to its availability through endogenous synthesis within the intestinal lumen. Specific taxa, including *Bifidobacterium* species and *Lactobacillus plantarum*, particularly in the presence of para-aminobenzoic acid, have been shown to produce folate, thereby supplementing host-derived pools [266]. This microbial contribution highlights a broader principle within the gut–brain axis. These findings suggest that micronutrient availability reflects both dietary intake and microbial metabolic activity. Following luminal production or ingestion, active forms of folate are absorbed predominantly in the small intestine through a combination of passive diffusion and carrier-mediated transport mechanisms [267]. In healthy adults, maintaining the recommended daily intake of approximately 400 μg/day is essential to support systemic metabolic demands [268].

The physiological importance of folate extends well beyond its classical metabolic roles. Deficiency states disrupt multiple interconnected pathways, leading to impaired nucleotide synthesis, reduced glutathione production, compromised immune cell function, and increased susceptibility to malignancy [268,269,270]. Within the context of the gut–brain axis, these effects are particularly relevant because they converge on processes, such as oxidative stress, inflammation, and cellular repair, that influence CNS stability. Notably, appropriate delivery of folate to the brain depends on intact BBB transport systems. Disruption of this interface has been linked to cerebral folate deficiency, a condition characterized by reduced levels of the biologically active form, 5-methyltetrahydrofolate, within the CNS despite normal systemic folate concentrations [271]. This compartmentalized deficiency underscores the importance of barrier integrity in mediating nutrient access to neural circuits. The gut microbiome may influence this process at multiple levels. By contributing to luminal folate production and potentially modulating intestinal absorption and barrier function, microbial communities may shape the effective delivery of folate to systemic and cerebral compartments. Although the precise dynamics of microbiome-dependent folate transport to the CNS remain to be fully elucidated, this interplay provides a plausible mechanistic framework linking microbial composition with neurochemical homeostasis. Moreover, because folate participates in methylation pathways that regulate gene expression, alterations in its availability may have downstream effects on neuronal development, synaptic plasticity, and neurotransmitter synthesis.

Evidence linking folate to epilepsy provides further support for its relevance within this framework. Clinically, folate deficiency has been associated with increased seizure susceptibility and severity, particularly in conditions characterized by impaired folate transport or metabolism. Cerebral folate deficiency, for example, is frequently accompanied by intractable seizures, and restoration of central folate levels through folinic acid supplementation leads to significant improvements in seizure control [272,273,274,275,276]. These observations support a causal role for central folate deficiency in seizure susceptibility, as restoration of folate levels improves seizure control. The improvement in seizure control underscores the importance of maintaining adequate central folate availability for stabilizing neuronal networks. In pediatric epilepsy cohorts, peripheral folate deficiency has been associated with increased seizure frequency and duration, whereas folic acid supplementation significantly reduced seizure activity [277], suggesting that folate availability may influence seizure outcomes. Similarly, clinical trials have reported reductions in seizure frequency following folic acid supplementation over defined treatment periods, although results in adult populations have shown greater variability [278]. These differences may reflect heterogeneity in underlying pathophysiology, dietary status, or ASM use across patient populations. The influence of folate on seizure susceptibility is further highlighted in conditions affecting its absorption and metabolism. Children with genetic defects impairing folate transport exhibit neurological manifestations including cognitive dysfunction, ataxia, and seizures, illustrating the consequences of disrupted folate homeostasis during critical developmental periods [279]. Conversely, excessive folate exposure may also carry risks. Elevated maternal folate supplementation has been associated with adverse neurodevelopmental outcomes, including memory deficits and increased seizure susceptibility in offspring, indicating that both deficiency and excess may perturb neural circuit stability [280], although the extent to which excess folate contributes to these outcomes remains unresolved.

Experimental models provide additional mechanistic insight. In rodent studies, folate administration reduces seizure-induced neuronal damage, an effect attributed in part to the preservation of BBB integrity and attenuation of oxidative stress [281,282]. These findings indicate that experimental folate administration can directly influence seizure-associated neuropathology. This neuroprotective effect aligns with the broader concept that folate exerts protective effects not only on neurons but also on the structural and metabolic environment that sustains neural function. Finally, the interaction between folate metabolism and antiseizure medications introduces an important clinical dimension. Several ASMs can reduce folate levels, thereby contributing to deficiency states and potentially influencing seizure control or comorbid outcomes [283]. This highlights the need for routine monitoring and targeted supplementation in appropriate patient populations, particularly when long-term pharmacotherapy is required.

Evidence linking folate to seizure susceptibility is supported by both observational and intervention studies, whereas evidence specifically connecting microbiota-derived folate to epilepsy remains comparatively limited. Together, these observations support folate as a key micronutrient within the gut–brain–microbiome axis. Importantly, both insufficient and excessive folate levels may contribute to neurological dysfunction, including seizure susceptibility, emphasizing the need for a balanced and context-dependent approach to folate supplementation.

#### 5.4.2. Pyridoxine (Vitamin B6)

Pyridoxine (vitamin B6) is an essential micronutrient obtained from both dietary sources and microbial synthesis within the gut. It exists in multiple forms, including pyridoxine, pyridoxal, and pyridoxamine, which are converted to the biologically active cofactor pyridoxal-5′-phosphate (PLP). Several gut bacterial taxa, including *Helicobacter pylori* (Proteobacteria), *Bifidobacterium longum* and *Collinsella aerofaciens* (Actinobacteria), and *Bacteroides fragilis* and *Prevotella copri* (Bacteroidetes), have been identified as contributors to pyridoxine production [284]. Typical daily intake recommendations range from approximately 1.6–2 mg/day [285].

PLP serves as a critical coenzyme in more than 100 enzymatic reactions and plays a central role in amino acid metabolism, myelin synthesis, and neurotransmitter production, underscoring its importance in CNS function [286]. Notably, PLP is required for the final step in the synthesis of key neurotransmitters, including dopamine and serotonin [287]. Circulating forms of pyridoxine are capable of crossing the BBB, enabling their contribution to cerebral metabolic processes [288]. Disruptions in pyridoxine availability or metabolism have significant neurological consequences. Deficiency has been associated with a range of disorders, including cognitive impairment, depression, Wernicke’s encephalopathy, and seizures [287]. Low pyridoxine levels may arise from multiple mechanisms, including inborn errors that impair its synthesis or activation, the accumulation of metabolites that inactivate its active cofactor form, the use of medications that interfere with its metabolism, and conditions that impair intestinal absorption [289]. In particular, several drugs, including enzyme-inducing antiepileptic agents, can alter pyridoxine metabolism, necessitating supplementation in certain clinical contexts to prevent adverse neurological outcomes [290].

The relevance of pyridoxine to epilepsy is well established. Both deficiency and inherited defects in pyridoxine metabolism are strongly linked to seizure susceptibility, including neonatal and convulsive disorders [291,292,293,294]. Clinically, reduced pyridoxine levels have also been observed in individuals with epilepsy and are associated with poorer seizure control [294]; however, these observations do not establish whether pyridoxine deficiency contributes to seizure severity. However, pyridoxine-dependent epilepsy represents a rare but important example of this relationship, in which accumulation of metabolic intermediates leads to inactivation of PLP, resulting in drug-resistant seizures in infancy that respond to pyridoxine supplementation [295]. This condition provides causal evidence linking disruption of pyridoxine metabolism to seizure generation, as restoration of pyridoxine availability suppresses otherwise drug-resistant seizures. Given that PLP is an essential cofactor in GABA synthesis, its depletion disrupts inhibitory neurotransmission, providing a mechanistic basis for seizure development.

Experimental studies further support a role for pyridoxine in modulating excitatory–inhibitory balance within the brain. For instance, at the molecular level, maternal pyridoxine deficiency has been associated with altered expression of GABA-related genes in offspring [296], suggesting a relationship between pyridoxine availability and inhibitory neurotransmission. Meanwhile supplementation increases expression of glutamic acid decarboxylase, a key enzyme in GABA synthesis, and decreases GABA transaminase activity in the hippocampus [297], indicating a role for pyridoxine in regulating inhibitory neurotransmission. Consistent with these effects on inhibitory neurotransmission, early experimental studies investigating pyridoxine and brain function, pyridoxine deficiency was shown to increase susceptibility to audiogenic and electroconvulsive seizures in mice, while supplementation exerted protective effects [298]. More recently, findings from disease-specific models have further highlighted the importance of pyridoxine availability in maintaining neuronal stability. In an *Aldh7a1*-deficient mouse model of pyridoxine-dependent epilepsy, reduced pyridoxine availability precipitated seizures, while pyridoxine treatment suppressed seizure activity and improved survival [299]. Supporting these in vivo observations, Girgis and colleagues demonstrated in primary mouse neuronal cultures that moderate reductions in pyridoxal-5′-phosphate activity increased neuronal firing and calcium signaling, resulting in a hyperexcitable neural phenotype [300].

Collectively, these findings underscore the importance of pyridoxine in maintaining neuronal function and highlight its dual role as both a metabolic cofactor and a therapeutic target in epilepsy. Ongoing efforts, including the development of international registries, continue to advance understanding of pyridoxine-dependent disorders and their clinical management, emphasizing the importance of this pathway in epilepsy research and treatment.

#### 5.4.3. Biotin (Vitamin B7)

In parallel with folate, biotin represents a microbiota-influenced micronutrient that links metabolic homeostasis with neural function, albeit through distinct biochemical and signaling pathways. Biotin is a water-soluble vitamin that, like most vitamins, cannot be synthesized by mammals and must therefore be obtained through a combination of dietary intake and microbial production within the gut ecosystem [301]. This dual origin underscores a recurring theme within the gut–brain axis: host micronutrient status reflects not only nutritional input but also the metabolic capacity and composition of the intestinal microbiota. Indeed, biotin-producing bacteria are distributed across multiple phylogenetic groups, including representatives of the Proteobacteria, Fusobacteria, and Bacteroidetes phyla [302], suggesting that microbial ecology may strongly influence luminal biotin availability.

Following production or ingestion, biotin participates in a range of essential cellular processes. Classically, it functions as a covalently bound cofactor for carboxylase enzymes, including acetyl-CoA carboxylase and pyruvate carboxylase, thereby supporting lipid metabolism, gluconeogenesis, and energy production [303]. However, as with other metabolites emerging from the gut–brain axis, its functional repertoire extends beyond metabolic catalysis. Biotin is increasingly recognized for its roles in cell signaling, epigenetic regulation, and chromatin remodeling, processes that collectively influence gene expression and cellular differentiation [303]. Importantly, biotin is actively transported across the BBB via the sodium-dependent multivitamin transporter [304] enabling it to contribute directly to central metabolic and regulatory pathways. An intake of approximately 30 µg/day is considered sufficient to support these physiological functions [267].

The gut microbiome likely influences biotin biology through its synthesis, availability, and potentially its absorption, while also shaping host biotin status via barrier interactions and metabolic competition. Disruptions in microbial composition, therefore, have the potential to alter both the quantity and functional impact of biotin available to host tissues. Indeed, recent experimental evidence demonstrates that biotin deficiency, whether induced by intestinal deletion of the biotin transporter or through dietary restriction, leads to a shift in microbial composition characterized by expansion of opportunistic taxa such as *Klebsiella*, *Enterobacter*, and *Helicobacter* [305]. Such changes highlight a bidirectional relationship in which biotin availability both reflects and influences the structure of the gut microbiome.

The relationship between biotin deficiency and seizures is most clearly illustrated in biotinidase deficiency, a genetic disorder caused by mutations in the gene encoding the enzyme responsible for recycling biotin [306]. In affected individuals, impaired biotin reutilization results in reduced availability of active biotin, despite normal dietary intake. Clinically, this condition presents with neurological and dermatological symptoms, including seizures, which are thought to arise from the accumulation of neurotoxic metabolites and disruption of metabolic homeostasis [306]. These observations underscore the importance of sustained biotin availability for maintaining neuronal stability. Reduced biotin levels have been reported in the cerebrospinal fluid of patients with epilepsy [307], providing associative evidence of altered central availability in disease states. Furthermore, long-term use of ASMs has been associated with decreased biotin levels [308,309], paralleling observations made with other micronutrients and reinforcing the importance of monitoring vitamin status in chronically treated patients. However, whether these ASM-induced deficiencies in biotin contribute to metabolic vulnerability and potentially influence seizure control or comorbid outcomes remains unclear. Therapeutically, biotin supplementation has demonstrated efficacy in specific contexts. Seizures associated with biotinidase deficiency are often refractory to conventional antiseizure therapies but respond dramatically to biotin replacement, with resolution of seizure activity following supplementation [310]. More broadly, studies have reported reductions in seizure frequency in children receiving biotin supplementation, although continued therapy appears necessary to maintain these effects [311]. These findings support a causal role for biotin deficiency in at least a subset of seizure disorders and highlight the potential for targeted supplementation strategies.

Experimental models further elucidate the relationship between biotin and neuronal excitability. In rodent studies, biotin deficiency accelerates seizure kindling and prolongs seizure duration, whereas supplementation reduces seizure severity, including tonic hindlimb extension in maximal electroshock models [312,313]. However, not all models recapitulate seizure phenotypes. For instance, genetic knockout of biotinidase in C57BL/6 mice results in neurological abnormalities such as demyelination and axonal degeneration without overt seizures [314,315]. These findings emphasize the importance of genetic background and experimental context in determining phenotypic outcomes, as well as the complexity of linking metabolic perturbations to seizure generation.

Together, these data support biotin as a mechanistically relevant micronutrient within the gut–brain–microbiome axis. As with folate, these observations underscore the importance of maintaining balanced vitamin homeostasis, as perturbations in either direction may contribute to neurological dysfunction, including seizure susceptibility.

#### 5.4.4. Cobalamin (Vitamin B12)

Cobalamin represents a critical micronutrient at the interface of microbial metabolism, systemic physiology, and CNS function [316]. Unlike many other vitamins, cobalamin is derived almost exclusively from dietary sources of animal origin, including meats, fish, eggs, dairy products, and certain fungi, while also being synthesized by a diverse array of gut microorganisms [316]. Microbial taxa implicated in cobalamin biosynthesis span a broad phylogenetic range, including *Acetobacterium*, *Bacillus*, *Clostridium*, *Corynebacterium*, *Escherichia*, *Eubacterium*, *Flavobacterium*, *Mycobacterium*, *Propionibacterium, Rhodopseudomonas*, *Streptococcus*, *Lactobacillus plantarum*, *and Bifidobacterium animalis* [316]. This widespread biosynthetic capacity suggests that microbial ecology may contribute to shaping cobalamin availability within the gut environment, although the extent to which microbially produced cobalamin contributes to host systemic stores remains an area of active investigation.

Following absorption in the ileum, cobalamin is transported in the circulation bound to transcobalamin II and subsequently delivered to peripheral tissues, including the CNS, where it enters cells via receptor-mediated endocytosis [317]. Through this transport pathway, cobalamin supports a range of essential physiological processes. Its functions extend from hematopoiesis and DNA synthesis to immune regulation and maintenance of myelin integrity, positioning it as a key regulator of both systemic and neural homeostasis [287,318]. Importantly, cobalamin has also been implicated in preserving BBB integrity, further reinforcing its relevance within the gut–brain axis. A daily intake of approximately 1–4 µg is generally sufficient to support these functions [319].

The mechanistic significance of cobalamin becomes particularly apparent in the context of neurological health. Deficiency is associated with disruptions in myelin synthesis and broader neurodegenerative processes that impair cognitive function and neural signaling [320,321]. Increasingly, cobalamin has also been implicated in protein homeostasis pathways relevant to neurodegeneration. For example, experimental evidence suggests that cobalamin may attenuate neurodegenerative processes by reducing tau aggregation and the formation of neurofibrillary tangles [322]. Clinical observations align with these findings, as decreased cobalamin levels have been reported in conditions such as Parkinson’s disease, whereas higher levels are associated with improved cognitive outcomes [320,321].

Within the gut–brain–microbiome axis, microbial contributions to cobalamin metabolism may add an additional layer of regulation. Microbial synthesis may influence luminal cobalamin availability and shifts in microbial composition could alter the balance between production, utilization, and host accessibility. Moreover, cobalamin-dependent metabolic pathways intersect with broader microbial processes, including amino acid and neurotransmitter metabolism, suggesting that microbiome composition may indirectly influence host neural signaling through cobalamin-sensitive pathways. Experimental studies further support a functional link between microbial cobalamin and neural signaling, as cobalamin-producing gut bacteria have been shown to modulate excitatory cholinergic signaling in C. elegans, illustrating a conserved interaction between microbial vitamin metabolism and host neural circuits [323].

Evidence linking cobalamin deficiency to epilepsy provides additional insight into its role in neuronal excitability. Inborn errors of intracellular cobalamin metabolism result in early-onset deficiency, leading to the accumulation of toxic metabolites and the development of neurological features including microencephaly and seizures [324]. Similarly, maternal dietary deficiency, such as that associated with vegetarian diets lacking adequate B12, has been linked to focal seizures in offspring [325]. However, cobalamin supplementation reduced seizure burden, although not fully reversing associated neurological deficits. In children with intractable epilepsy, long-term use of ASMs has been associated with reduced cobalamin levels, suggesting that pharmacological treatment itself may contribute to micronutrient depletion [326,327]. In certain cases, supplementation alongside ASMs has been reported to improve epilepsy-associated symptoms [326,327], indicating that restoration of cobalamin homeostasis may support neurological function in selected patient populations. Experimental evidence provides additional mechanistic support, as cobalamin administration has been shown to suppress penicillin-induced epileptiform activity in rodent models [328], suggesting that cobalamin availability can direct effect on neuronal excitability. The interaction between cobalamin status and the gut microbiome introduces a further dimension of complexity. While supplementation does not appear to significantly alter microbial composition in healthy states, it can induce measurable shifts under inflammatory conditions such as colitis, indicating that its microbial effects are context-dependent [329].

#### 5.4.5. Riboflavin (Vitamin B2)

Riboflavin is an essential water-soluble vitamin that mammals are unable to synthesize and must therefore obtain from dietary sources, including animal products, vegetables, legumes, and plant-derived foods [330]. Complementing this dietary contribution, numerous gut bacteria, particularly *Bifidobacterium* and *Lactobacillus* species, can produce riboflavin, and advances in microbial fermentation technologies have further expanded their availability for nutritional and therapeutic applications [284,331]. As such, riboflavin availability reflects a convergence of diet, microbial biosynthesis, and host absorption.

At the molecular level, riboflavin serves as a precursor for key coenzymes, including flavin adenine dinucleotide and flavin mononucleotide, which are essential for redox reactions and mitochondrial energy metabolism. Through these roles, riboflavin contributes to cellular bioenergetics, oxidative phosphorylation, and metabolic homeostasis. However, consistent with emerging paradigms in gut–brain biology, its functional repertoire extends beyond classical metabolism. Experimental evidence indicates that riboflavin exerts antioxidant and anti-inflammatory effects [332,333], positioning it as a modulator of oxidative stress and immune signaling, two processes intimately linked to neuronal excitability and the pathophysiology of epilepsy.

Riboflavin absorption occurs predominantly in the small intestine, where it is transported across epithelial cells and enters circulation via specific carrier-mediated pathways. Transport into the bloodstream is facilitated by riboflavin transporters 1 and 2 located on the basolateral membrane [334]. Once in circulation, riboflavin can cross the BBB to be taken up by neurons and astrocytes [335], thereby directly contributing to central metabolic processes and redox balance. Maintaining an adequate intake, approximately 1.3 mg/day in adults, is essential to sustain these functions [336].

Within the gut ecosystem, riboflavin also participates in a bidirectional relationship with the microbiome. Microbial synthesis contributes to luminal vitamin pools, while riboflavin availability can influence microbial metabolic activity. Although supplementation exerts limited effects on overall microbial composition under basal conditions, it enhances butyrate production [337], a key SCFA with established roles in gut–brain communication. Given that butyrate has been implicated in the modulation of GABAergic signaling and neuronal excitability [338,339], this interaction suggests that riboflavin may indirectly influence CNS function through microbiome-mediated metabolic pathways.

Riboflavin deficiency is associated with a range of neurological and metabolic disturbances, reflecting its central role in cellular energy metabolism and redox regulation. Deficiency states have been linked to impaired nerve function, migraine, abnormal brain development, and neurodegeneration, as well as rare genetic disorders such as riboflavin transporter deficiency (Brown–Vialetto–Van Laere syndrome) [340]. These observations emphasize the importance of efficient riboflavin transport and utilization in maintaining neuronal integrity. Clinical studies have further shown that supplementation can improve motor symptoms in Parkinson’s disease patients with low riboflavin levels [341], supporting its role in neuronal maintenance and resilience.

Evidence linking riboflavin to epilepsy, while limited, provides several mechanistic and clinical insights. In a clinical case of epilepsy associated with short-chain acyl-CoA dehydrogenase deficiency, seizures that were initially unresponsive to valproate improved following riboflavin addition to the treatment regimen [342], suggesting that restoration may influence seizure outcomes in selected metabolic disorders. This finding suggests that riboflavin may enhance mitochondrial function and metabolic stability in conditions where energy metabolism is compromised, although its direct influence on seizure susceptibility remains unclear. Mechanistic studies further support a potential anticonvulsant role. Riboflavin has been shown to suppress endogenous glutamate release by reducing presynaptic voltage-gated calcium currents in cortical nerve terminals [343]. Given the central contribution of glutamatergic signaling to neuronal excitation and seizure generation, this effect provides a biologically plausible mechanism through which riboflavin may modulate excitability and seizure threshold. Clinical observations also highlight the interaction between riboflavin status and antiseizure therapies. Reduced riboflavin levels have been reported in pregnant women with epilepsy receiving ASMs [344], suggesting that long-term pharmacological treatment may contribute to depletion of this micronutrient.

While direct causal effects on seizure control remain unclear, such findings highlight the importance of monitoring vitamin status in populations exposed to chronic ASM therapy. Despite these observations, human studies directly linking riboflavin supplementation to improved seizure outcomes remain limited.

#### 5.4.6. Vitamin K

Vitamin K is a fat-soluble micronutrient that exists primarily as phylloquinone (vitamin K1) and menaquinones (vitamin K2). Phylloquinone is obtained predominantly from green leafy vegetables and other plant-derived foods, whereas menaquinones are produced largely by the gut microbiota, including species such as *Eubacterium lentum* and *Bacteroides* spp. [285,345,346]. Recommended daily intake generally ranges from 70–300 µg/day [347]. The absorption of vitamin K is mediated by multiple transport systems, including Niemann–Pick C1-like 1 protein, scavenger receptor class B type I, and cluster determinant 36, facilitating its uptake within the intestine and subsequent distribution throughout the body [348].

Beyond dietary intake, the gut microbiota represents a significant source of vitamin K2 in the form of menaquinones. Numerous intestinal bacterial species synthesize menaquinones, and experimental evidence suggests that microbially derived vitamin K can be absorbed by the host and contribute to circulating vitamin K pools [285]. Consequently, alterations in microbial community composition may influence vitamin K availability. Indeed, disruption of menaquinone-producing bacteria, including during antibiotic exposure, has been associated with reduced endogenous vitamin K production and an increased risk of vitamin K insufficiency [349]. Although the quantitative contribution of microbial vitamin K synthesis to overall vitamin K status remains incompletely resolved, these findings provide a mechanistic link between the gut microbiome and vitamin K-dependent physiological processes [285]. While vitamin K is best known for its indispensable role in coagulation through the activation of clotting factors, accumulating evidence indicates that its physiological functions extend well beyond hemostasis to include cardiovascular health, bone metabolism, and cognitive function [350]. In addition, vitamin K has emerged as an important regulator of immune and inflammatory pathways. Experimental studies demonstrate that vitamin K exerts both direct and indirect anti-inflammatory, antioxidant, and immunomodulatory effects, suggesting that it may influence diverse physiological systems, including the CNS [351].

Consequently, inadequate vitamin K status has been associated with a range of pathological outcomes, including cognitive dysfunction [352]. A potential role for vitamin K in brain function is further supported by its unique distribution within the nervous system. Notably, menaquinone-4 (MK-4), a vitamin K2 isoform synthesized from phylloquinone, is the predominant form detected in the brain [353]. Evidence from experimental studies suggests that vitamin K contributes to neuronal survival, as alterations in vitamin K status influence brain lipid composition and pathways important for maintaining neuronal integrity [354]. Experimental studies further indicate that vitamin K exerts neuroprotective effects by limiting oxidative stress-induced neuronal and oligodendrocyte injury, supporting a role in maintaining cellular redox homeostasis [355].

Evidence directly linking vitamin K to epilepsy remains limited but intriguing. In the 6-Hz psychomotor seizure model, vitamin K3 derivatives reduced seizure activity, providing early evidence that vitamin K-related pathways may influence neuronal excitability [356]. Similarly, vitamin K-related quinone compounds have demonstrated anticonvulsant efficacy in the maximal electroshock seizure model, a finding that further supports a potential role for vitamin K-associated signaling pathways in seizure modulation [357]. Although the precise mechanisms underlying these effects remain unclear, the established roles of vitamin K in regulating oxidative stress, neuronal survival, and membrane lipid metabolism suggest that it may contribute to the maintenance of neural network stability through multiple complementary mechanisms. Clinical evidence is currently sparse; however, children with febrile seizures have been reported to exhibit higher circulating concentrations of vitamin K1 and vitamin K2 compared with healthy controls [358], highlighting the need for further investigation into the complex relationship between vitamin K metabolism and seizure susceptibility.

Vitamin K may also be relevant to epilepsy through indirect clinical mechanisms. Maternal use of certain antiseizure medications during pregnancy can impair fetal vitamin K status, increasing the risk of coagulopathy and severe hemorrhagic complications in newborns [359]. Importantly, focal seizures are among the major neurological manifestations of neonatal vitamin K deficiency, emphasizing the critical importance of maintaining adequate vitamin K levels during early development. Collectively, these findings suggest that although evidence directly linking vitamin K to epilepsy remains relatively limited compared with other vitamins, emerging data supports further exploration of vitamin K-dependent mechanisms at the intersection of the gut microbiome, neuroinflammation, and neuronal excitability.

#### 5.4.7. Vitamin D

In contrast to the water-soluble B vitamins discussed above, vitamin D represents a fat-soluble micronutrient and prohormone that integrates environmental exposure, dietary intake, and systemic signaling to influence CNS function. Vitamin D is synthesized endogenously in the skin following exposure to ultraviolet radiation, with additional contributions from dietary sources such as fatty fish, egg yolk, and certain mushrooms [360]. This dual origin reinforces its unique positioning within the gut–brain axis, where nutrient availability reflects not only dietary intake and microbial influences but also environmental factors.

Following synthesis or ingestion, vitamin D undergoes hepatic conversion to 25-hydroxyvitamin D [25(OH)D], the primary circulating form, which can cross the BBB [361]. Within the CNS, 25(OH)D is subsequently converted by neurons and glial cells into its active form, 1,25-dihydroxyvitamin D [1,25(OH)_2_D], which exerts its effects through the vitamin D receptor (VDR) [361]. Notably, VDR expression is widespread across brain regions implicated in seizure generation and modulation, including the cerebellum, hippocampus, hypothalamus and substantia nigra [362]. This anatomical distribution suggests that vitamin D signaling may directly influence neural circuits associated with excitability and seizure susceptibility.

Beyond its classical role in calcium and bone homeostasis, vitamin D exhibits a broad range of biological activities relevant to CNS health. It participates in anti-inflammatory and antioxidant pathways, regulates intracellular calcium dynamics, modulates reactive oxygen species, and influences cell cycle processes, neurodevelopment, and brain volume [363]. These functions intersect with key mechanisms implicated in epileptogenesis, including neuroinflammation, oxidative stress, and dysregulated calcium signaling, positioning vitamin D as a critical regulator of neuronal stability. Serum levels ≥30 ng/mL are generally considered necessary to support these diverse physiological functions [360].

Although gut microbes do not synthesize vitamin D, the relationship between vitamin D and the microbiome is nevertheless bidirectional. Vitamin D signaling influences microbial community composition, and supplementation has been shown to shift microbiota profiles in multiple contexts. For example, increases in beneficial taxa such as Ruminococcaceae and *Akkermansia* have been reported, alongside reductions in certain Firmicutes genera [364]. Other studies describe broader compositional shifts, including changes in Firmicutes, Bacteroidetes, Proteobacteria, and Actinobacteria, with reductions in taxa such as Veillonellaceae and Oscillospiraceae following supplementation [365].

Deficiency of vitamin D is relatively common, particularly in older adults, and has been associated with a wide range of autoimmune, neuropsychiatric, and neurodegenerative disorders [361]. Mechanistically, vitamin D deficiency impacts multiple neurotransmitter systems, including dopaminergic, glutamatergic, and GABAergic pathways [366], thereby influencing the balance of excitatory and inhibitory signaling within neural circuits. Given the central role of this balance in seizure susceptibility, disruptions in vitamin D signaling may contribute to neuronal hyperexcitability through both direct neurochemical and indirect immune-mediated mechanisms.

Evidence linking vitamin D to epilepsy remains heterogeneous but increasingly suggestive of a modulatory role. Case reports have described seizure activity associated with severe hypocalcemia secondary to vitamin D deficiency, with resolution following vitamin D and calcium supplementation [367], supporting a causal role for severe vitamin D deficiency in seizure generation in this clinical context. More robust clinical evidence is emerging from interventional studies. A recent randomized clinical trial demonstrated that long-term vitamin D_3_ supplementation reduced seizure frequency, particularly bilateral tonic–clonic seizures, with efficacy correlating with achieving serum 25(OH)D levels above 30 ng/mL [368]. These findings support a dose-dependent relationship between vitamin D status and seizure control. Additional clinical observations implicate vitamin D in treatment responsiveness. Cross-sectional analyses indicate that vitamin D levels may vary according to the number and type of ASMs used [369], suggesting that both disease severity and pharmacological treatment may influence vitamin D status. However, these observations do not establish whether altered vitamin D status influences seizure control. Small pilot trials in adults have reported reductions in seizure activity following vitamin D_3_ supplementation [370,371], although results are not consistent across studies, with some studies showing no significant benefit. These discrepancies likely reflect heterogeneity in patient populations, baseline vitamin D status, and study design.

Experimental models provide further mechanistic support for the role of vitamin D in regulating neuronal excitability. Genetic deletion of the vitamin D receptor provides causal evidence that VDR-mediated signaling contributes to maintaining neural stability, as receptor deletion increases seizure susceptibility [372]. At the molecular level, VDR-dependent transcription regulates proteins involved in excitability, including components of GABAergic signaling pathways [372], thereby linking vitamin D activity directly to inhibitory neurotransmission. Further support comes from pharmacological studies demonstrating that administration of active vitamin D metabolites increases electroconvulsive threshold and potentiates the anticonvulsant effects of ASMs [373]. Similarly, earlier work in rodent models showed that vitamin D_3_ administration elevates hippocampal seizure threshold [374], reinforcing its role in modulating seizure susceptibility.

#### 5.4.8. Vitamin E

In contrast to the vitamin pathways described above, many of which are partially supported by microbial biosynthesis, vitamin E represents a microbiota-influenced, but not microbiota-derived, fat-soluble micronutrient that exerts its effects primarily through antioxidant and membrane-stabilizing mechanisms. Vitamin E, particularly in its biologically active form α-tocopherol, is obtained predominantly from dietary sources such as vegetable oils, soybeans, nuts, and green vegetables [375]. Although gut microbes do not synthesize vitamin E, they participate in shaping the intestinal environment in which vitamin E is absorbed and utilized.

At the molecular level, vitamin E functions as a major lipid-soluble antioxidant, protecting cellular membranes from oxidative damage by scavenging reactive oxygen species. Beyond this classical role, it also contributes to immune regulation, maintenance of intestinal barrier integrity, and modulation of inflammatory signaling pathways [375]. The recommended daily intake for vitamin E is approximately 15 mg/day, reflecting its essential role in maintaining systemic and cellular homeostasis [376].

Despite the absence of direct microbial synthesis, vitamin E engages in dynamic interactions with the gut microbiome. Vitamin E supplementation has been shown to influence microbial composition in a context-dependent manner [377]. Moreover, experimental models demonstrate that vitamin E administration can ameliorate microbiome disruption and intestinal barrier dysfunction, particularly in inflammatory states such as colitis [102]. These findings suggest that vitamin E contributes to gut homeostasis not only through its direct effects on epithelial integrity and immune signaling but also through indirect modulation of microbial communities.

Preclinical studies provide some evidence linking vitamin E to epilepsy. In PTZ-induced seizure models, vitamin E supplementation reduces oxidative stress and mitigates seizure-associated neuropathology [378], supporting its role as a neuroprotective agent. Similarly, in KA-induced temporal lobe epilepsy models, pretreatment with vitamin E attenuates both the severity and frequency of seizures [379]. Vitamin E treatment has also been shown to prevent KA-induced dendritic spine loss [379], a structural indicator of synaptic dysfunction and seizure-associated network remodeling that contributes to neuronal hyperexcitability. In addition, post-seizure administration of the active form of vitamin E, α-tocopherol, confers neuroprotective effects by reducing neuronal cell death and limiting neurodegeneration following epileptogenic injury [380], supporting a neuroprotective role for vitamin E after seizure-related damage. Collectively, these results highlight the capacity of vitamin E to influence both acute and chronic aspects of seizure-related pathology through stabilization of neuronal membranes and suppression of oxidative damage.

### 5.5. Polyphenol-Derived Metabolite: S-Equol

Building on the role of vitamins and microbially derived metabolites, polyphenols represent an additional class of diet-dependent compounds whose biological activity is largely revealed through microbial transformation. These plant-derived molecules are abundant in fruits, vegetables, tea, chocolate, and wine and are increasingly recognized as modulators of the gut–brain–microbiome axis [381]. Their effects are mediated, in part, through antioxidant and anti-inflammatory properties, as well as through their capacity to influence microbial composition and function [381]. Among polyphenols, flavonoids have received particular attention for their roles in neural differentiation, survival, and neuroinflammatory regulation [382]. Among these, isoflavones are phytoestrogens structurally related to estrogen. They are of particular interest due to their capacity to generate bioactive metabolites through gut microbial metabolism. S-equol represents an example of such a microbial-derived gut metabolite. It is generated exclusively from the isoflavone daidzein, a dietary component of soy, through the action of gut bacteria [383]. Notably, although equol exists as two enantiomers (R- and S-equol), only the S-enantiomer is produced in humans following microbial metabolism [384]. This dependence on microbial conversion introduces an important determinant of bioavailability, as only a subset of individuals harbors microbial communities capable of producing S-equol. Once formed, S-equol exhibits high bioavailability, with approximately 50% circulating in an unbound form, thereby facilitating receptor interaction in peripheral tissues and the CNS [385].

**Figure 2:**
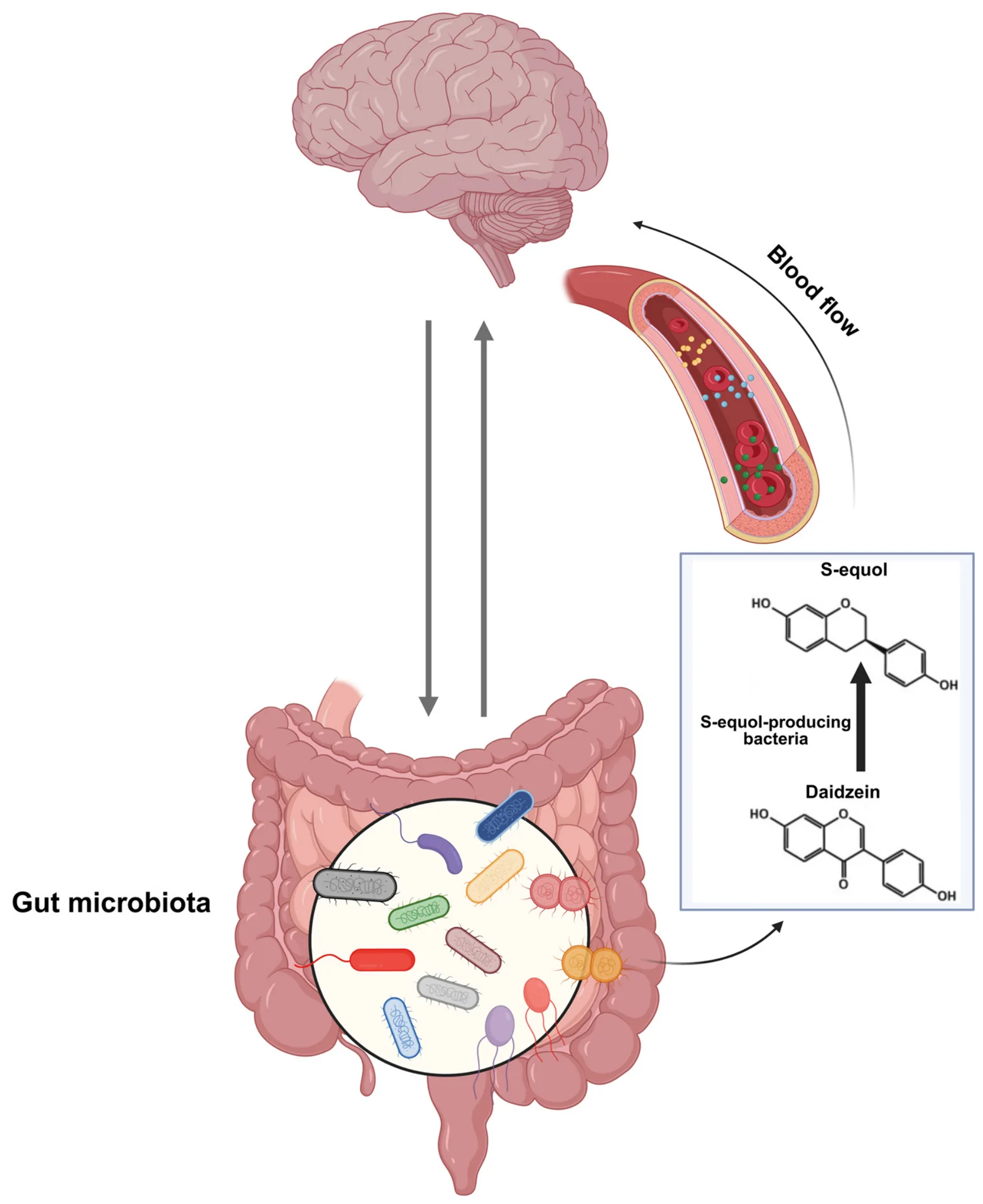
Schematic illustrating the production of the bioactive metabolite S-equol by specific gut bacteria through the conversion of the dietary isoflavone daidzein. S-equol enters the circulation, crosses the BBB, and may influence central nervous system function via the gut–brain axis. S-equol is presented as an illustrative example of a microbiota-derived bioactive metabolite. Created in BioRender. Guo, X. (2026); https://biorender.com/n5jzch5 (accessed on 21 July 2026).

Following production in the gut, S-equol demonstrates efficient permeability across both the intestinal barrier and the BBB (Figure 2), enabling it to exert direct effects on central neural circuits [386]. Mechanistically, S-equol functions as a phytoestrogen with high affinity for estrogen receptor beta (ERβ), exhibiting preferential binding compared to estrogen receptor alpha (ERα) [387]. ERβ is widely expressed in brain regions relevant to seizure generation, including the cortex, hippocampus, and cerebellum, positioning S-equol to directly influence neuronal signaling networks. In addition to classical nuclear receptor signaling, S-equol activates membrane-associated estrogen receptors, including the G protein-coupled receptor GPER1/GPR30, which is expressed in several CNS regions and contributes to rapid signaling cascades [388]. S-equol also modulates ion channels, including large-conductance Ca^2+^-activated K^+^ channels (BK). In particular, it enhances the activity of BK channels, increasing outward potassium currents that promote membrane repolarization and reduce neuronal firing in the sub-micromolar to low-micromolar range [389]. This dual capacity of modulating both receptor signaling and ion channel function positions S-equol as a multifaceted regulator of neuronal excitability.

The broader functional properties of S-equol further support its relevance to CNS biology. It exhibits potent antioxidant activity, with greater efficacy than many other isoflavones, and exerts anti-inflammatory and neuroprotective effects across multiple experimental systems [390,391,392]. These properties intersect closely with pathways implicated in neurodegeneration and neuropsychiatric disorders. Indeed, clinical and preclinical studies indicate that S-equol may influence neurocognitive function, mood regulation, and risk of neurodegenerative diseases. Higher dietary intake of soy isoflavones has been associated with reduced risk of cognitive impairment and dementia [385], although the reported findings did not establish whether S-equol itself causally mediates the protective association. In other studies, supplementation has been shown to improve mood and cognitive outcomes in peri- and postmenopausal populations [393,394,395,396,397,398,399]. At the cellular level, S-equol attenuates microglial activation, reduces apoptosis and excitotoxicity, and promotes dendritic growth and astrocyte-mediated repair processes [400,401,402,403,404,405], collectively supporting neuronal resilience.

Several of these mechanisms converge on pathways directly relevant to epilepsy. ERβ signaling within the hippocampus and cortex has been implicated in regulating the balance between excitation and inhibition, and selective modulation of this receptor has been associated with anticonvulsant effects, although these responses may be sex-dependent and context-specific [406,407,408]. In parallel, activation of BK channels by S-equol provides a direct mechanism for dampening neuronal hyperexcitability by facilitating repolarization and limiting intracellular calcium accumulation, consistent with observations that loss-of-function BK channel mutations increase seizure susceptibility [387,389,406,409].

The involvement of GPER1 adds an additional signaling dimension. Experimental studies demonstrate that disruption of GPER1 signaling alters seizure dynamics. For example, knockdown of GPER1 reduces latency to seizure onset and increases seizure severity in rodent models [192]. Reduced GPER1/PKA signaling has also been observed in epilepsy-associated pathologies, including focal cortical dysplasia type IIb and tuberous sclerosis complex, with associations to increased seizure frequency [410]. Pharmacological modulation of this receptor produces complex effects: GPER1 agonists attenuate neuronal injury and aberrant circuit remodeling in certain models, such as reducing mossy fiber sprouting in temporal lobe epilepsy [411], yet may also exacerbate seizure susceptibility in other settings, including PTZ-kindling paradigms [412].

At the level of neuronal injury and excitotoxicity, S-equol directly attenuates glutamate-induced toxicity and protects against hypoxia-induced cell death [401,402], aligning with established mechanisms of seizure-induced neuronal damage. Importantly, recent work has linked S-equol production to microbiome-dependent modulation of seizure susceptibility. In the TMEV model of epilepsy, S-equol-producing bacteria were depleted in mice that developed seizures, providing associative evidence linking loss of S-equol-producing taxa with seizure susceptibility [413]. In the same model, exogenous S-equol administration reduced neuronal hyperexcitability in affected animals [413,414], supporting a functional role for this metabolite in modulating seizure-related neuronal activity. These findings provide evidence for a functional axis linking gut microbial metabolism, S-equol production, and neuronal excitability.

S-equol thus emerges as a microbiome-dependent neuromodulator that integrates dietary polyphenol intake, microbial metabolism, and host signaling pathways. Its ability to engage estrogen receptors, modulate BK channels, attenuate oxidative stress, and regulate neuroinflammatory responses provides multiple convergent mechanisms through which it may influence seizure susceptibility. The dependence of its production on specific microbial communities further underscores the importance of interindividual variability in gut microbiota composition. Future studies aimed at defining the causal impact of S-equol supplementation or microbiome modulation on seizure outcomes will be essential for determining its translational potential in epilepsy.

## 6. Conclusions

Over the past several decades, advances in microbiome research have reshaped our understanding of how peripheral biological systems influence CNS function. Accumulating evidence from both preclinical and clinical studies now firmly positions the gut microbiota as a key regulator within the gut–brain axis, with emerging relevance to epilepsy and seizure susceptibility. Alterations in microbial composition have been observed in both drug-responsive and drug-resistant epilepsy, suggesting that the microbiome may participate not only in disease pathophysiology but also in therapeutic response.

Despite this progress, important gaps remain in defining the mechanistic pathways that connect microbial composition to neuronal excitability. As highlighted throughout this review, the gut–brain axis operates through a network of interacting systems, neural, endocrine, immune, and metabolic, rather than a single linear pathway. Within this framework, gut-derived metabolites, micronutrients, and signaling molecules act as intermediaries that translate microbial activity into host physiological responses. However, the precise integration of these pathways and the extent to which specific metabolites exert causal effects on seizure generation or suppression remain incompletely resolved.

A central challenge in the field lies in the inherent complexity and variability of microbiomes. Differences across experimental systems, including animal models, diet, age, sex, and analytical approaches, contribute to heterogeneity in findings. At the same time, the microbiome itself introduces an additional layer of variability, as individual differences in microbial composition influence the production and activity of key metabolites such as SCFAs, neurotransmitter-related compounds, vitamins, and polyphenol-derived molecules like S-equol. This variability complicates efforts to identify consistent biomarkers or therapeutic targets but also underscores the potential for personalized, microbiome-informed approaches to epilepsy management. Interpretation of the current literature is further complicated by methodological limitations across both preclinical and clinical studies. These challenges include differences in microbiome sequencing and analytical methodologies, small sample sizes, and potential confounding factors such as diet, medication exposure, and host-specific factors. Consequently, findings should be interpreted with appropriate caution until validated in larger, standardized and reproducible studies.

The recognition that gut microbes contribute to CNS function represents a significant conceptual shift. It highlights that neural homeostasis depends not only on intrinsic cellular mechanisms but also on the metabolic outputs of a dynamic microbial ecosystem. Within this context, an important unresolved question is whether microbial alterations act as drivers of epileptogenesis or primarily modulate disease severity and progression. Determining whether specific microbial changes increase or decrease seizure susceptibility, and under what conditions, remains a priority for future research. Equally critical is the need to delineate how distinct classes of gut-derived molecules, including SCFAs, neurotransmitter analogs, vitamins, and polyphenol metabolites, interact within shared signaling networks that regulate excitability.

From a translational perspective, microbial-derived metabolites hold promise as both biomarkers and therapeutic targets. Their accessibility in peripheral compartments and their mechanistic links to CNS function make them attractive candidates for diagnostic and intervention strategies. However, substantial challenges remain. Current approaches are largely correlative, and efforts to selectively manipulate disease-associated microbes or metabolic pathways are still in early stages. Future studies will require greater methodological standardization, larger and more diverse cohorts, and rigorous control of potential confounding variables to improve reproducibility and strengthen confidence in reported associations. Progress in this area will require more precise tools to establish causality, including controlled dietary interventions, targeted microbial modulation, and mechanistic studies that connect peripheral metabolic changes to defined neural circuit outcomes.

The findings summarized in this review illustrate the breadth of interactions between the gut microbiome and seizure-related pathways, spanning metabolism, immune regulation, neurotransmission, and barrier integrity. Continued investigation of these interactions is essential to move beyond descriptive associations toward mechanistic clarity. As this field advances, integrating microbiome profiling with functional and translational studies will be critical for determining how gut-derived signals can be harnessed to improve outcomes in epilepsy.

## Data Availability

No new data were created or analyzed in this study. Data sharing is not applicable to this article.

## Abbreviations

The following abbreviations are used in this manuscript:

ACTH	adrenocorticotropic hormone
CNS	central nervous system
ASMs	anti-seizure medications
BDNF	brain-derived neurotrophic factor
BBB	blood–brain barrier
CRH	corticotropin-releasing hormone
DNA	deoxyribonucleic acid
L-DOPA	L-3,4-dihydroxyphenylalanine
DMNV	dorsal motor nucleus of the vagus
DVC	dorsal vagal complex
EECs	enteroendocrine cells
ERβ	estrogen receptor beta
FFAR	free fatty acid receptor 2, 3
GABA	γ-aminobutyric acid
HPA	hypothalamic–pituitary–adrenal
5-HIAA	5-hydroxyindoleacetic acid
5-HTP	5-hydroxytryptophan
HCAR1/GPR81	hydroxycarboxylic acid receptor 1
IL-6	interleukin 6
KA	kainic acid
KD	ketogenic diet
LDH	lactate dehydrogenase
JB-1	*Lactobacillus rhamnosus*
BK	large-conductance calcium-activated K^+^ channels
MK-4	menaquinone-4
mGluRs	metabotropic glutamate receptors
MCTs	monocarboxylate transporters
PTZ	pentylenetetrazole
PLP	pyridoxal-5′-phosphate
5-HT	serotonin
SCFAs	short-chain fatty acids
SUDEP	sudden unexpected death in epilepsy
TMEV	Theiler’s murine encephalomyelitis virus
TCA	tricarboxylic acid
TPH1	tryptophan hydroxylase 1
TH	tyrosine hydroxylase
TNF-α	tumor necrosis factor-alpha
VDR	vitamin D receptor
